# Cost-Effectiveness of 2023-2024 COVID-19 Vaccination in US Adults

**DOI:** 10.1001/jamanetworkopen.2025.23688

**Published:** 2025-08-07

**Authors:** Lisa A. Prosser, Megan Wallace, Angela M. Rose, Kerra Mercon, Cara B. Janusz, Acham Gebremariam, David W. Hutton, Andrew J. Leidner, Fangjun Zhou, Ismael R. Ortega-Sanchez, Danielle Moulia, Ruth Link-Gelles, Sharon Saydah, Melisa Shah, Jamison Pike

**Affiliations:** 1 Department of Health Management and Policy, School of Public Health, University of Michigan, Ann Arbor; 2 Susan B. Meister Child Health Evaluation and Research Center, University of Michigan Medical School, Ann Arbor; 3 Centers for Disease Control and Prevention, Atlanta, Georgia; 4 Department of Implementation Science, Wake Forest University School of Medicine, Winston-Salem, North Carolina; 5 Public Health Service, Commissioned Corps, Rockville, Maryland

## Abstract

**Importance:**

Evidence is needed on the economic favorability of a 2023-2024 COVID-19 vaccination program to support policy decisions on recommendations for COVID-19 vaccination.

**Objective:**

To measure the cost-effectiveness of vaccination with a 2023-2024 COVID-19 mRNA vaccine in adults aged 18 years or older compared with no updated vaccination (with a 2023-2024 vaccine).

**Design, Setting, and Participants:**

This decision analytic modeling study used a simulation model to compare outcomes for hypothetical cohorts of vaccinated and unvaccinated, immunocompetent adults stratified by age (18-49 years, 50-64 years, and ≥65 years). Parameters were drawn from primary and published data to represent characteristics of the US adult population. The interventions included vaccination with a 2023-2024 mRNA vaccine against COVID-19–associated illness compared with no updated vaccination (with a 2023-2024 vaccine) using a 1-year analytic time frame. A phase 2 analysis also considered an intervention strategy of vaccination with an additional dose of 2023-2024 COVID-19 mRNA vaccine.

**Main Outcome and Measure:**

The incremental cost-effectiveness ratio as reported in 2023 US dollars per quality-adjusted life-year.

**Results:**

Vaccination averted substantial numbers of cases, hospitalizations, intensive care unit stays, and deaths across age groups. For adults aged 18 to 49 years and 50 to 64 years, vaccination yielded incremental cost-effectiveness ratios of $115 588 and $25 787 per quality-adjusted life-year gained, respectively. For individuals aged 65 years or older, vaccination was cost saving. Sensitivity analyses indicated that the results were most sensitive to the cost per vaccine dose, vaccine effectiveness, and probability of hospitalization. In phase 2 analyses, an additional dose strategy was only economically favorable in higher-risk scenarios for individuals aged 65 years or older.

**Conclusions and Relevance:**

In this modeling study, economic favorability of COVID-19 vaccination varied by age. Cost-effectiveness results for individuals in the 2 older age groups were favorable and generally robust to changes in parameter inputs, while results for the younger age group were sensitive to parameter input changes. As the evidence base for COVID-19 vaccination and burden of illness evolves, it may be important to continue to update and revise the economic evaluation of vaccination. The Advisory Committee on Immunization Practices considered these results in its decision to recommend vaccination with the 2023 to 2024 COVID-19 mRNA vaccines.

## Key Points

**Question:**

What is the cost-effectiveness of vaccination with a 2023-2024 COVID-19 mRNA vaccine in adults aged 18 years or older compared with no updated vaccination?

**Findings:**

This decision analytic modeling study in a simulated cohort projected that cost-effectiveness for a 2023-2024 COVID-19 mRNA vaccine using base case assumptions was cost saving for individuals older than 65 years, $25 787 per quality-adjusted life-year gained for those aged 50 to 64 years, and $115 588 per quality-adjusted life-year gained for those aged 18 to 49 years. Results were robust to changes in parameter inputs for the older age groups but sensitive to inputs for the younger age group.

**Meaning:**

These findings suggest that vaccination is economically attractive for older adults but only under certain conditions for younger adults.

## Teaser

This qualitative analytic modeling study measures the cost-effectiveness of the 2023-2024 COVID-19 mRNA vaccine in US adults aged 18 years or older compared with no updated vaccination.

## Introduction

Despite prevention and control efforts, COVID-19 still causes substantial morbidity and mortality in adults.^1^ During 2020 to 2021, 2 mRNA vaccine products were authorized by the US Food and Drug Administration (FDA) for emergency use to prevent severe COVID-19 illness.^2^ Updated monovalent 2023-2024 COVID-19 vaccines were approved by the FDA in September 2023.^3^ In September 2023 and February 2024, the Centers for Disease Control and Prevention’s (CDC’s) Advisory Committee on Immunization Practices (ACIP) considered recommendations for the use of 2023 to 2024 COVID-19 vaccines.^4^

The objectives of this study were to help inform ACIP recommendations on seasonal COVID-19 vaccination by (1) estimating annual disease burden and health care utilization associated with COVID-19 illness and 2023-2024 COVID-19 vaccination and (2) projecting the cost-effectiveness of vaccination with a 2023-2024 COVID-19 mRNA vaccine in adults aged 18 years or older compared with no updated vaccination (with 2023-2024 vaccine). A second phase of the study was conducted in February 2024 to inform ACIP recommendations for an additional midyear dose by evaluating the incremental health benefits and cost-effectiveness of a second dose of a 2023-2024 COVID-19 mRNA vaccine for US adults.

## Methods

### Overall Approach

This decision analytic modeling study used a simulation model to project health and economic outcomes for COVID-19 illness and vaccination. The study was deemed exempt from institutional review board review and informed consent by University of Michigan policy as it did not involve human participant research. This study followed the Consolidated Health Economic Evaluation Reporting Standards (CHEERS) reporting guideline.

Intervention strategies were (1) vaccination against COVID-19 with a 2023-2024 mRNA vaccine and (2) no updated vaccination. During the fall and winter of 2023-2024, 2 COVID-19 mRNA vaccines were available in the US. For simplicity and because these vaccines have similar attributes, this model evaluated COVID-19 vaccination assuming a generalized mRNA vaccine as the intervention. The comparison group of no updated vaccination represented immunocompetent individuals with no updated COVID-19 vaccination for 2023 to 2024, consistent with recent epidemiologic data that showed immunity across the population due to vaccination with a previous COVID-19 vaccine formulation, experience of COVID-19 illness, or both.^5^ The target population included US adults (immunocompetent), stratified by age as follows: 18 to 49 years, 50 to 64 years, and 65 years or older. The time horizon for the analysis was 1 year but accounted for costs and health impacts beyond 1 year associated with premature death or long-term sequelae following hospitalization due to COVID-19 illness. The simulation model was programmed in TreeAge Pro 2023, release 2 (TreeAge Software).

**Figure 1.**
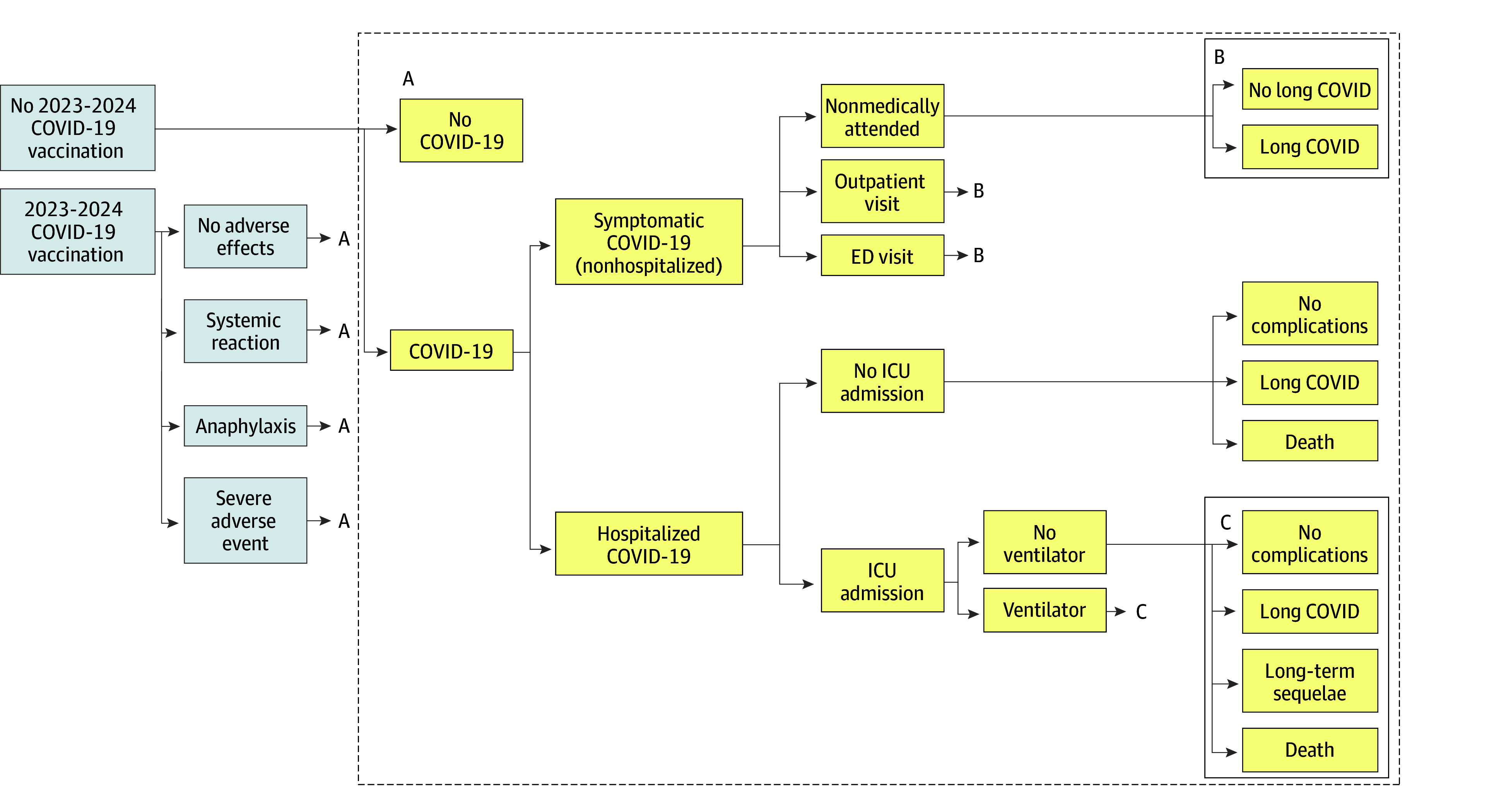
Detailed Model Schematic Asymptomatic COVID-19 was not considered in the model. No complications indicates the absence of long COVID (defined as ongoing COVID-19 symptoms for an average period of 5 months that limited daily function), long-term sequelae, or death following a hospitalization. Emergency department (ED) visit and COVID-19 testing have associated probabilities and costs but were not included as separate health states. Long-term sequelae included complications following an intensive care unit (ICU) stay for COVID-19 (eg, acute kidney injury, ischemic stroke, pulmonary fibrosis) and were mutually exclusive to long COVID.

Two identical cohorts were run through the model, one in which all individuals receive a single dose of 2023-2024 mRNA vaccine in the fall and one in which they did not (Figure 1). In both, an individual had some probability of remaining disease free or experiencing symptomatic COVID-19 illness (Table 1). If individuals experienced COVID-19 illness, they could have an episode of nonhospitalized illness or become hospitalized. Individuals with symptomatic illness could also incur an outpatient or emergency department visit. If hospitalized, the individual could require intensive care unit (ICU) stay (with or without ventilator assistance). All individuals who experienced a COVID-19 illness-related outpatient visit or hospitalization were at risk for long COVID (defined as ongoing COVID-19 symptoms for an average period of 5 months that limited daily function) (Table 1; eTable 1 in Supplement 1). Individuals with an ICU stay could also experience long-term permanent sequelae or death. Simulated individuals accumulated costs and losses in quality of life associated with each health state. For vaccination, the model included associated adverse events, including systemic reactions, anaphylaxis, and myocarditis (Figure 1; eTable 2 in Supplement 1). These events also incurred costs and losses in quality of life.

**Table 1.** Selected Model Inputs of Probabilities for COVID-19–Related Events, Costs, Vaccine Effectiveness, and Quality-of-Life Adjustments^a^

Input and age group	Base case (range for sensitivity analysis)	Source
**Phase 1 input values**		
Probability inputs		
Annual probability of symptomatic COVID-19^b^		
18-49 y	0.3145 (0.2858-0.3444)	R. Wiegand, PhD, email, May 26, 2023^c^
50-64 y	0.2841 (0.2438-0.3274)	
≥65 y	0.3339 (0.2312-0.4510)	
Probability of long COVID given outpatient visit or hospitalization^d^^,^^e^		
≥18 y	0.072 (0.058-0.091)	Montoy et al,^6^ 2023
Annual probability of hospitalization		
18-49 y	0.00144 (0.00080-0.00204)	COVID-NET,^1^ October 2022 to March 2023
50-64 y	0.00335 (0.00216-0.00479)	
≥65 y	0.01453 (0.00967-0.02090)	
Probability of ICU admission given hospitalization		
18-49 y	0.123 (0.119-0.145)	COVID-NET,^1^ October 2022 to March 2023
50-64 y	0.200 (0.178-0.208)	
≥65 y	0.144 (0.138-0.163)	
Probability of ventilator assistance given ICU admission^f^		
18-49 y	0.525 (0.472-0.577)	COVID-NET,^1^ October 2022 to March 2023
50-64 y	0.488 (0.445-0.532)	
≥65 y	0.386 (0.342-0.432)	
Probability of death given hospitalization (no ICU)		
18-49 y	0.006 (0.002-0.008)	COVID-NET,^1^ October 2022 to March 2023
50-64 y	0.009 (0.007-0.016)	
≥65 y	0.030 (0.022-0.035)	
Probability of death given ICU admission (no ventilator assistance)		
18-49 y	0.024 (0.003-0.040)	COVID-NET,^1^ October 2022 to March 2023
50-64 y	0.047 (0.026-0.077)	
≥65 y	0.166 (0.144-0.233)	
Probability of death given ICU admission (ventilator assistance)		
18-49 y	0.284 (0.213-0.368)	COVID-NET,^1^ October 2022 to March 2023
50-64 y	0.379 (0.301-0.435)	
≥65 y	0.628 (0.476-0.637)	
Vaccine effectiveness		
Vaccine effectiveness against nonhospitalized symptomatic illness^g^		
≥18 y	0.269 (0.088-0.418)	Link-Gelles,^7,8^ 2023
Vaccine effectiveness against hospitalization^h^		
≥18 y	0.269 (0.088-0.418)	Link-Gelles,^7,8^ 2023
Vaccine effectiveness against hospitalization in ICU^i^		
≥18 y	0.403 (0.191-0.671)	Link-Gelles,^8^ 2023
Vaccine effectiveness against death^i^		
≥18 y	0.403 (0.191-0.671)	Link-Gelles,^8^ 2023
Costs		
Vaccine, 1 dose^j^		
All manufacturers	$120 ($30-$200)	Kates et al,^9^ 2023
Vaccine administration		
Per dose^k^	$20.33 ($18.07-$26.58)	Centers for Medicare & Medicaid Services^10^
Recipient time by setting^l^		
Mass vaccination	0.195 (0.000-0.390)	Prosser et al,^11^ 2008
Physician office	1.190 (0.170-2.000)	Prosser et al,^11^ 2008
Pharmacy	0.250 (0.083-0.500)	Prosser et al,^11^ 2008, and expert opinion
Outpatient visit, direct medical		
18-49 y	$372 ($370-$375)	F. Zhou, PhD, unpublished data, 2025^m^
50-64 y	$380 ($377-$384)	
≥65 y	$391 ($386-$396)	
Long COVID, direct medical^d^^,^^n^		
Outpatient visits	$1091 ($1018-$1165)	Pike et al,^12^ 2023^o^
Hospitalization, direct medical		
18-49 y	$32 514 ($28 505-$36 523)	F. Zhou, PhD, unpublished data, 2025^m^
50-64 y	$32 854 ($31 450-$34 258)	
≥65 y	$20 648 ($20 295-$21 000)	
ICU stay (no ventilator assistance), direct medical		
18-49 y	$37 159 ($30 116-$44 203)	F. Zhou, PhD, unpublished data, 2025^m^
50-64 y	$46 727 ($40 269-$53 186)	
≥65 y	$23 220 ($22 408-$24 032)	
ICU stay (with ventilator assistance), direct medical		
18-49 y	$245 432 ($168 362-$322 503)	F. Zhou, PhD, unpublished data, 2025^m^
50-64 y	$169 189 ($140 250-$198 129)	
≥65 y	$55 257 ($50 705-$59 809)	
Quality-of-life adjustments		
Illness (QALY loss per episode)^p^		
Symptomatic	0.006 (0.004-0.008)	S. Johnson, MPH, email, May 26, 2023^q^
Hospitalization	0.027 (NA)	
Critical illness	0.054 (NA)	
Long COVID^d^^,^^r^	0.067 (0.038-0.088)	
Systemic reaction (QALY loss)^s^		
≥18 y	0.0004 (0.0003-0.0005)	Assumption
**Updated input values for phase 2 model**		
Annual probability of hospitalization		
18-49 y	0.00044 (0.00001-0.00204)	COVID-NET,^1^ October 2022 to September 2023^t^
50-64 y	0.00155 (0.00041-0.00479)	
≥65 y	0.00790 (0.00245-0.02090)	
Seasonality-adjusted vaccine impact^u^		
Symptomatic illness and hospitalization		
1-Dose strategy		
18-49 y	0.360 (0.140-0.475)	COVID-NET,^1^ October 2022 to September 2023; Link-Gelles,^7,8^ 2023
50-64 y	0.357 (0.141-0.475)	
≥65 y	0.347 (0.134-0.468)	
2-Dose strategy		
18-49 y	0.431 (0.175-0.506)	
50-64 y	0.436 (0.180-0.510)	
≥65 y	0.434 (0.179-0.509)	
ICU and death		
1-Dose strategy		
18-49 y	0.462 (0.287-0.664)	COVID-NET,^1^ October 2022 to September 2023; Link-Gelles,^7,8^ 2023
50-64 y	0.457 (0.282-0.666)	
≥65 y	0.451 (0.272-0.666)	
2-Dose strategy		
18-49 y	0.546 (0.382-0.667)	
50-64 y	0.549 (0.386-0.669)	
≥65 y	0.549 (0.386-0.669)	
Vaccine dose cost^v^^,^^w^		
All manufacturers	$124 ($30-$200)	Centers for Disease Control and Prevention,^13^ 2025

Abbreviations: ICU, intensive care unit; NA, not available; QALY, quality-adjusted life-year.

^a^ A complete list of model inputs is provided in eTables 1 to 4 in Supplement 1.

^b^ Using data from December 2022 to May 2023.

^c^ Unpublished data from HEROS-RECOVER.

^d^ Long COVID defined as ongoing COVID-19 symptoms for an average period of 5 months that limited daily function.

^e^ Average prevalence of head, ear, eye, nose, throat; constitutional; pulmonary; musculoskeletal; cognitive; and fatigue symptoms.

^f^ Adjusted for ventilator assistance cases not in the ICU.

^g^ Using bivalent booster data from September 2022 to May 2023. Range includes minimum and maximum from both data sources, applying minimum/conservative assumption (vaccine effectiveness [VE] at 180 days = 0) and maximum/optimistic assumption (VE at 180 days = VE at 365 days); assumed same as hospitalization.

^h^ Bivalent booster data from September 2022 to May 2023. Range includes minimum and maximum from both data sources, applying minimum/conservative assumption (VE from 180 to 365 days = 0) and maximum/optimistic assumption (VE from 180 to 365 days = VE at 180 days [eg, no further waning beyond 180 days]).

^i^ Bivalent booster data from September 2022 to May 2023.

^j^ Lower bound reflects price of bivalent boosters as of March 2023.

^k^ *Common Procedural Terminology* code 90471.

^l^ Proportion by setting: mass vaccination = 0.100; physician office visit = 0.256; pharmacy = 0.644.

^m^ Unpublished data from Merative Marketscan.

^n^ Adjusted to 2023 US dollars using a blended 2020/2021 adjuster. Proportion of individuals with long COVID who have cognitive symptoms and/or fatigue. Fifty percent productivity loss also applied to 51% of individuals with long COVID. Duration of illness assumed to be 150 days.

^o^ Five-month costs derived from 3- and 6-month costs using linear interpolation.

^p^ Quality-adjusted life-year loss per person. Derived using the ratio of ICU to hospitalization QALY loss (2 times) applied to the EQ-5D survey of hospitalization QALY loss.

^q^ Unpublished data from the SARS-CoV-2 Epidemiology and Response in Children, Prospective Assessment of COVID-19 in a Community, and Coronavirus Household Evaluation and Respiratory Testing studies.

^r^ Quality-adjusted life-year loss adjusted to 5-month duration.

^s^ Assumption, QALY loss equal to QALY loss associated with 1 day of COVID-19 illness, EQ-5D survey.

^t^ Inputs from COVID-NET data October 2022 to September 2023 adjusted by the probability of COVID-19 given hospitalization in October to December (0.709), January to March (0.719), April to June (0.729), and July to September (0.789). Upper limit from October 2022 to March 2023 was unadjusted.

^u^ Vaccine effectiveness for a generalized 2023-2024 COVID-19 mRNA vaccine was estimated using unpublished data on the 2022-2023 bivalent vaccine from 2 multicenter Centers for Disease Control and Prevention–funded VE platforms: the Virtual SARS-CoV-2, Influenza, and Other Respiratory Viruses Network and the Investigating Respiratory Viruses in the Acutely Ill Network. Estimates for the 2023-2024 monovalent vaccine were not yet available. The waning of vaccine effectiveness over a 1-year duration was combined with monthly rates of hospitalization and critical illness to estimate seasonality-adjusted vaccine impact.

^v^ Lower bound reflects price of bivalent boosters as of March 2023.

^w^ Costs and adverse events associated with vaccination were 2 times the parameter inputs used in the 1-dose strategy.

In a phase 2 analysis evaluating the cost-effectiveness of delivering an additional dose of a 2023-2024 COVID-19 mRNA vaccine, the model was updated to include a third intervention strategy comparing no updated vaccination, a single dose of the updated vaccine, and a single dose plus an additional midyear dose (2 doses) of the updated vaccine (eFigure 1 in Supplement 2). The timing of the additional dose was modeled to occur 6 months following the initial fall 2023-2024 dose.

### Epidemiologic Inputs

Model parameters were derived using published and unpublished data along with expert panel opinion. Due to the rapidly evolving evidence base for COVID-19 illness and vaccination, many parameters in the model used unpublished data that reflected the best available evidence. The probability of symptomatic illness was based on the most recent 6 months of data from the HEROES-RECOVER study, a network of prospective cohorts among frontline workers across 6 states with weekly SARS-CoV-2 testing (R. Wiegand, PhD, email, May 26, 2023) from December 2022 to May 2023 (Table 1). Six-month rates were converted to annual probabilities and stratified by age group. The probability of an outpatient or emergency department visit due to COVID-19 was derived using Merative Marketscan, a large representative claims database (F. Zhou, PhD, email, August 29, 2023). The annual probability of hospitalization due to COVID-19 illness stratified by age was derived using the most recent 6 months of data available (October 2022 to March 2023) from the COVID-19 Hospitalization Surveillance Network (COVID-NET), a CDC-funded network that monitors laboratory-confirmed, COVID-19–associated hospitalizations.^1^ The conditional probability of requiring ICU care, ventilator assistance, or death given hospitalization were also derived from COVID-NET data (December 2021 to March 2023).^1^ Parameters for long COVID were estimated using published data,^6^ assuming a median symptom duration of 5 months, and were not stratified by age.

In phase 2 analyses for February 2024, hospitalization rates were updated with more recent COVID-NET data (October 2022 to September 2023) to reflect observed declines (Table 1).^1^ Hospitalization rates included in phase 2 were also adjusted to exclude patients with incidental COVID-19 who were hospitalized for other conditions.

### Vaccine Effectiveness and Vaccination-Associated Adverse Events

Vaccine effectiveness (VE) for a generalized 2023-2024 COVID-19 mRNA vaccine was estimated using unpublished data on the 2022-2023 bivalent vaccine from 2 multicenter CDC-funded VE platforms: the Virtual SARS-CoV-2, Influenza, and Other Respiratory Viruses Network and the Investigating Respiratory Viruses in the Acutely Ill Network.^7,8^ Using VE estimates for 180 days following vaccination from both platforms, we calculated the area under the curve using 3 scenarios to extrapolate 180-day VE to 1 year: (1) an optimistic scenario that assumed the VE observed between 120 and 180 days remained for the duration of the year (upper bound); (2) a conservative scenario that assumed VE waned to 0 at 181 days, sustained through to 1 year (lower bound), and (3) a midpoint scenario that assumed linear waning from the VE observed at 180 days until the year’s end (base case) (eFigure 1 in Supplement 1). Given similarities in recent VE estimates in the US across the spectrum of illness severity, an assumption was made to apply the VE for uncomplicated hospitalization to symptomatic illness, as VE was not available for this outcome.^7,8^

Probabilities of vaccination-associated adverse events (eg, systemic reactions, anaphylaxis, myocarditis or pericarditis) were estimated using FDA fact sheets,^14,15^ published data,^16,17,18^ and expert opinion. A probability of dying of either vaccination-associated anaphylaxis or myocarditis was also included (eTable 2 in Supplement 1).

For phase 2 analyses, VE waning over a 1-year duration^7,8^ was combined with monthly rates of hospitalization and critical illness to derive seasonality-adjusted vaccine impact to replace the phase 1, nonadjusted VE estimates previously described. This new parameter better reflects vaccine impact by incorporating vaccination timing and variation in monthly infection rates, meaning that vaccine impact is highest when the months with the highest infection rates also have the highest VE (ie, prior to any VE waning) (Table 1; eFigure 2 in Supplement 2). Seasonality-adjusted vaccine impact estimates were calculated to model an initial dose of vaccine that was given in October with seasonality (eg, monthly infection rate) based on the most current year of infection data. Alternate patterns of seasonality (with infection peaks in different seasons) were considered in scenario analyses.

### Costs

Costs included direct medical costs, time costs associated with obtaining health care services, and productivity losses due to COVID-19 illness or vaccination-associated adverse events. Direct medical costs associated with a case of symptomatic COVID-19 illness that incurred an outpatient visit or hospitalization were estimated using unpublished Merative Marketscan data and stratified by age (Table 1). Posthospitalization direct medical costs within 1 year, as well as costs of long-term permanent sequelae, were derived from published estimates.^19,20,21,22,23^ Direct medical costs associated with an episode of long COVID were derived from published estimates using IQVIA PharMetrics Plus data.^12^ Productivity losses were estimated from unpublished data using time lost by patients and/or caregivers during illness and were included for each outcome, varying with illness severity (eTable 3 in Supplement 1).

Costs of vaccination included the direct medical costs of the updated 2023-2024 mRNA vaccine dose^9^ and administration,^10^ as well as recipient time costs (Table 1).^11^ Time costs varied by vaccination setting. Proportion of vaccinations delivered by setting was based on recent survey data.^24^ Costs of vaccination-associated adverse events included direct medical costs and productivity losses. All costs were adjusted 2023 US dollars using the Gross Domestic Product Implicit Price Deflator.^25^

### Quality-of-Life Adjustments

Quality-of-life adjustments for COVID-19 illness and vaccination-associated adverse events were incorporated as losses in quality-adjusted life-years (QALYs) (Table 1; eTable 4 in Supplement 1). Quality-adjusted life-year losses for COVID-19 illnesses and hospitalizations were calculated using primary data collected via 3 longitudinal surveys implemented by the CDC using the EQ-5D survey (Table 1). Quality-adjusted life-year losses for higher-severity hospitalizations were estimated using primary data from 2 health preference surveys fielded in 2021 and 2023 with time-tradeoff questions.^26^ Quality-of-life adjustments for long-term sequelae after hospitalization and vaccination-associated adverse events were based on published data and expert opinion.^27,28,29^

### Analysis Plan

For phase 1 analyses, the primary outcome was the incremental cost-effectiveness ratio (ICER) in US dollars per QALY gained, comparing vaccination with a single dose of updated 2023-2024 COVID-19 mRNA vaccine with no updated vaccination. This metric measures the additional investment in vaccination, or cost saving, associated with a change in health benefits, and accounts for offsets due to averted illness. In phase 2 analyses, the primary outcome (US dollars per QALY gained) included 3 intervention strategies: (1) no 2023-2024 vaccine (no updated vaccination), (2) 1 dose of the 2023-2024 mRNA vaccine (single-dose strategy), and (3) 2 doses of the 2023-2024 mRNA vaccine (2-dose, or additional dose, strategy).

Secondary outcomes included disaggregated health and economic outcomes stratified by intervention strategy, age subgroups (18-49 years, 50-64 years, and ≥65 years), cases of COVID-19 illness, hospitalizations, deaths, total costs, and total QALYs. The analytic time horizon was 1 year, accounting for costs, productivity losses, and quality adjustments beyond 1 year for long-term sequelae and deaths (discounted by 3%).

The analysis used both health care sector and limited societal perspectives, with the (limited) societal perspective as the primary perspective (health care sector results available in eTable 5 in Supplement 1). Additional details are available in an impact inventory (eTable 6 in Supplement 1).

Uncertainty analyses (one-way sensitivity and scenario analyses) were performed across plausible ranges for parameter input values. One-way sensitivity analyses were performed across all variables using a range of parameter input values. Scenario analyses explored the impact of additional uncertainty for VE and probability of symptomatic illness, higher probability of hospitalization and critical care (proxy for high-risk group), vaccination setting, and inclusion of unrelated health care costs on ICERs.

To directly reflect the results that were presented to the ACIP for decision-making in September 2023 (phase 1) and February 2024 (phase 2), phase 1 results are presented separately and use a separate set of inputs from the additional dose phase 2 analysis. Results for an updated, 1-dose analysis using phase 2 inputs are included in eTables 1 to 4 in Supplement 2.

## Results

### Phase 1: Single Dose of an Updated Vaccine

#### Cases Averted

For a hypothetical cohort of immunocompetent US adults, projected hospitalizations, ICU stays, and deaths averted increased with increasing age. Hospitalizations averted with vaccination ranged from 39 per 100 000 for individuals aged 18 to 49 years to 391 per 100 000 for those aged 65 years or older. Deaths averted with vaccination ranged from 1.4 per 100 000 for individuals aged 18 to 49 years to 43.4 per 100 000 for those aged 65 years or older. Projected cases averted were similar across age groups, ranging from 7642 to 8982 per 100 000 (eTable 7 in Supplement 1).

#### ICERs

Vaccination with a 2023-2024 COVID-19 mRNA vaccine yielded additional health benefits across all age groups, yet the magnitude of benefits and the societal investment required varied by age. For individuals aged 18 to 49 years and 50 to 64 years, vaccination required additional investment for the incremental health benefits gained, yielding $115 588 and $25 787 per QALY, respectively. For individuals aged 65 years or older, vaccination yielded cost saving compared with no vaccination (Table 2).

**Table 2.** Total Projected Costs and QALYs, Incremental Costs and QALYs, and ICERs Per 100 000 (Societal Perspective)

Age group and strategy	COVID-19 cases, No.	Hospitalizations, No.	ICU admissions, No.	Adverse events, No.	Deaths, No.	Projected costs, $	Incremental costs, $	QALYs, No.		ICER, $/QALY gained
								Projected	Incremental	
**Phase 1, single-dose analysis (September 2023)**										
18-49 y										
No updated 2023-2024 COVID-19 vaccination	31 450	144	17.7	NA	3.6	$19 233 487	NA	2 020 707	NA	NA
Vaccination (2023-2024 COVID-19 vaccine)^a^	22 990	105	10.6	10 602	2.2	$29 350 294	$10 116 807	2 020 794	88	$115 588
50-64 y										
No updated 2023-2024 COVID-19 vaccination	28 410	335	67.1	NA	16.4	$38 575 162	NA	1 227 583	NA	NA
Vaccination (2023-2024 COVID-19 vaccine)^a^	20 768	245	40.0	10 601	9.9	$42 124 948	$3 549 786	1 227 721	138	$25 787
≥65 y										
No updated 2023-2024 COVID-19 vaccination	33 390	1453	209.3	NA	109.4	$64 248 755	NA	651 995	NA	NA
Vaccination (2023-2024 COVID-19 vaccine)^a^	24 408	1062	124.9	13 701	66.0	$59 885 706	−$4 363 049	652 355	360	CS
Pooled ≥18 y										
Vaccination (2023-2024 COVID-19 vaccine)^a^	NA	NA	NA	NA	NA	NA	NA	NA	NA	$33 437
**Phase 2, additional dose analysis (February 2024)** ^b^										
18-49 y										
No updated 2023-2024 vaccination	31 450	44	5.4	NA	1.1	$11 257 637	NA	2 020 761	NA	NA
Vaccination, 1 dose (2023-2024 COVID-19 vaccine)	20 128	28	2.9	10 602	0.6	$24 917 936	$13 660 299	2 020 844	84	$163 255
Vaccination, 2 doses (2023-2024 COVID-19 vaccine)	17 895	25	2.5	21 203	0.5	$41 985 675	$17 067 739	2 020 857	13	$1 317 714
50-64 y										
No updated 2023-2024 COVID-19 vaccination	28 410	155	31.0	NA	7.6	$23 321 232	NA	1 227 698	NA	NA
Vaccination, 1 dose (2023-2024 COVID-19 vaccine)	18 268	100	16.8	10 600	4.3	$32 269 460	$8 948 228	1 227 809	111	$80 427
Vaccination, 2 doses (2023-2024 COVID-19 vaccine)	16 023	87	14.0	21 201	3.6	$48 293 374	$16 023 914	1 227 830	21	$777 612
≥65 y										
No updated 2023-2024 COVID-19 vaccination	33 390	790	113.8	NA	59.5	$39 025 205	NA	652 338	NA	NA
Vaccination, 1 dose (2023-2024 COVID-19 vaccine)	21 804	516	62.5	13 700	35.1	$41 993 902	$2 968 697	652 587	249	$11 936
Vaccination, 2 doses (2023-2024 COVID-19 vaccine)	18 899	447	51.3	27 401	29.5	$55 944 019	$13 950 117	652 642	55	$255 122

Abbreviations: CS, cost saving; ICER, incremental cost-effectiveness ratio; ICU, intensive care unit; NA, not applicable; QALY, quality-adjusted life-year.

^a^ Vaccination against COVID-19 with a generalized 2023-2024 mRNA vaccine.

^b^ An initial dose in the fall followed by an additional dose approximately 6 months following the initial dose.

#### Uncertainty Analyses

**Figure 2.**
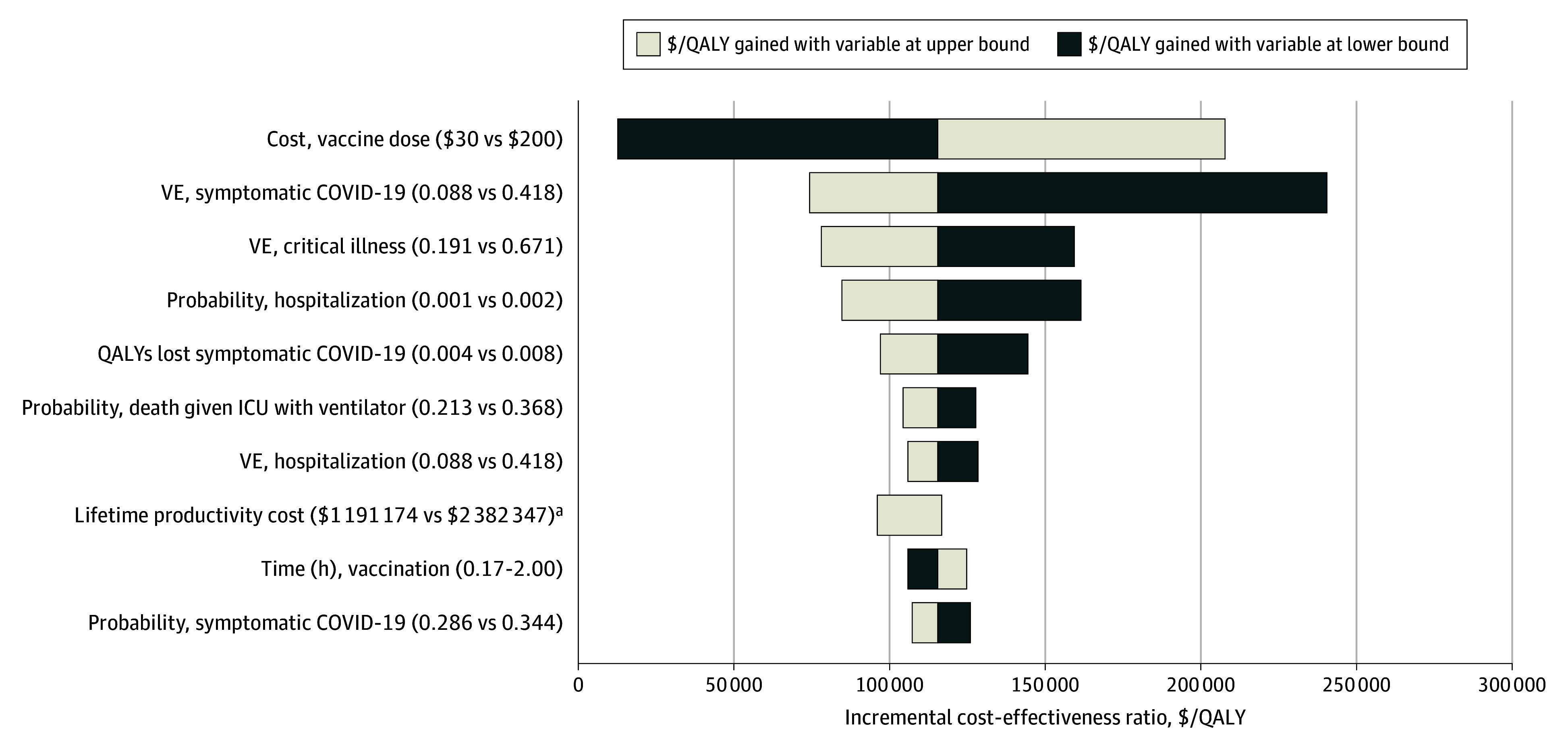
One-Way Sensitivity Analyses for Most Influential Parameters, 2023-2024 COVID-19 Vaccination Compared With No Updated Vaccination, Adults Aged 18 to 49 Years The base case incremental cost-effectiveness ratio was $115 588. Critical illness indicates intensive care unit (ICU) stays and deaths. QALY indicates quality-adjusted life-year; VE, vaccine effectiveness. ^a^Lower input was equal to the base case.

One-way sensitivity analyses across all parameters were most sensitive to the cost per vaccine dose, VE, and probability of hospitalization, with variation in these parameters yielding ICERs of more than $150 000 per QALY among individuals aged 18 to 49 years (Figure 2). One-way sensitivity analyses for older ages were robust to changes in the input parameters, yielding ICERs from cost saving to $83 902 per QALY (eTable 8 and eFigure 2 in Supplement 1).

Additional scenario analyses explored the uncertainty associated with VE, symptomatic illness, and probabilities of hospitalization and critical illness using values beyond those in the primary analysis (eTables 9-11 in Supplement 1). Setting VE against all end points to all upper or all lower bounds yielded ratios ranging from $45 376 to $435 886 per QALY gained for individuals aged 18 to 49 years, from cost saving to $199 830 per QALY gained for those aged 50 to 64 years, and from cost saving to $51 782 per QALY gained for those aged 65 years or older (eTable 9 in Supplement 1).

Varying the probability of symptomatic illness from 50% to 10% was influential for individuals aged 18 to 49 years (range, $75 905-$229 724 per QALY gained) but yielded smaller changes to ICERs in those aged 50 to 64 years (range, $10 123-$48 937 per QALY gained). The ICERs for individuals aged 65 years or older remained cost saving for this range of the probability of symptomatic illness (eTable 10 in Supplement 1).

Increasing the probability of hospitalization from 2 to 4 times the base case as a rough proxy for individuals at higher risk of COVID-19 complications yielded substantially more favorable ICERs across all ages, from cost saving to $71 487 per QALY gained (Table 3). Excluding long COVID from the analysis (given the higher level of uncertainty for this end point) yielded an ICER of $133 402 per QALY gained for individuals aged 18 to 49 years, $33 724 per QALY gained for those aged 50 to 64 years, and remaining cost saving for those aged 65 years or older (eTable 12 in Supplement 1). Assuming a lower VE against symptomatic COVID-19 illness yielded a substantially higher ICER of $239 913 per QALY gained for individuals aged 18 to 49 years, but was less influential for those aged 50 to 64 years ($54 411 per QALY gained) or 65 years or older (cost saving) (eTable 13 in Supplement 1). Inclusion of unrelated health care costs did not appreciably change ICERs, yielding an ICER of $119 225 per QALY gained for individuals aged 18 to 49 years, $36 524 per QALY gained for those aged 50 to 64 years, and $2403 per QALY gained for those aged 65 years or older from the societal perspective (eTables 14 and 15 in Supplement 1).

**Table 3.** Scenario Analyses of Probability of Hospitalization and Critical Care, QALY Gained, and Societal Perspective, Phase 1

Age group	ICER, $/QALY gained			
	Base case	2 Times base case value	3 Times base case value	4 Times base case value
**Probability of hospitalization^a^**				
18-49 y	$115 588	$51 978	$14 541	CS
50-64 y	$25 787	CS	CS	CS
≥65 y	CS	CS	CS	CS
**Probability of critical care**				
18-49 y	$115 588	$71 487	$42 307	$21 570
50-64 y	$25 787	CS	CS	CS
≥65 y	CS	CS	CS	CS

Abbreviations: CS, cost saving; ICER, incremental cost-effectiveness ratio; QALY, quality-adjusted life-year.

^a^ Adjusted risk of hospitalization by underlying condition: chronic obstructive pulmonary disease, 0.9; history of stroke, 0.9; coronary artery disease, 1.3; asthma, 1.4; hypertension, 2.8; obesity, 2.9; diabetes, 3.2; chronic kidney disease, 4.0; and severe obesity, 4.4.^30^

### Phase 2: Additional Dose of an Updated Vaccine

The ICERs for a 2-dose compared with no updated vaccination strategy were $1 317 714 per QALY gained for individuals aged 18 to 49 years, $777 612 per QALY gained for those aged 50 to 64 years, and $255 122 per QALY gained for those aged 65 years or older (Table 1). In uncertainty analyses, a 2-dose strategy was not cost saving in any of the scenario analyses using a societal perspective (eTable 3 and eFigure 3 in Supplement 2). Scenarios that were a proxy for a higher-risk subgroup of individuals older than 65 years were associated with favorable ICERs. Vaccination with an additional dose for individuals older than 65 years also yielded ICERs at $127 105 per QALY gained or lower for a cost per dose of less than $50 (eTable 3 and eFigure 3 in Supplement 1).

## Discussion

This decision analytic modeling study shows that a single dose of a 2023-2024 COVID-19 mRNA vaccine averted substantial morbidity and mortality across age groups; however, the economic attractiveness of vaccination varied considerably by age. The ICERs for vaccination of individuals aged 50 to 64 years and 65 years or older with a single dose were robust to changes in parameter inputs across plausible ranges in all but 1 scenario (VE set to lower bounds for all end points). In contrast, ICERs for individuals aged 18 to 49 years with a single dose were very sensitive to changes in parameter inputs, yielding more favorable ICERs in scenarios with higher VE, higher risk of hospitalization and critical illness, and lower vaccine dose cost. The ACIP considered these results in its decision to recommend vaccination with a 2023-2024 COVID-19 vaccine for all individuals aged 6 months or older in September 2023.^4^ Using updated hospitalization data available for the February 2024 analysis, the ICERs associated with an additional dose strategy were less favorable for adults aged 18 to 49 years or 50 to 64 years across plausible parameter ranges. An additional dose of 2023-2024 COVID-19 mRNA vaccine was not supported by the economic evidence for individuals aged 18 to 64 years but became more economically favorable for higher-risk individuals aged 65 years or older.

At the time of the September 2023 ACIP meeting, there was only one other analysis of 2023-2024 COVID-19 vaccination available as a preprint.^31^ Using a different modeling approach (ie, compartmental susceptible-exposed-infected-recovered model), this study reported a pooled ICER for all adults older than 18 years of $2100 per QALY gained. Key differences from our analysis were the inclusion of transmission effects, more favorable VE, and higher rates of hospitalization. Notwithstanding, results from the previous study’s sensitivity analysis that used a lower initial VE were similar to those in our analysis, with an ICER of $27 000 per QALY gained compared with $33 000 per QALY gained for the adult population in the base case for our analysis.

This report presents results from the first 2 phases of an ongoing analysis. Pediatric age groups were excluded from the initial phases of the analysis due to the scarcity of data available to develop stable parameter inputs for either the 5- to 11-year or 12- to 17-year age groups. Compared with other vaccine-preventable respiratory illnesses, the evidence base even for adults was scant. This consideration was key in the decision during the design of the analysis to forego a probabilistic analysis until further evidence was available. Given the high level of uncertainty for most parameters in the model, incorrect parameterization of these distributions may give incorrect results and (paradoxically) a false sense of certainty in the confidence of the results.

### Limitations

This analysis had several limitations. First, the analysis relied primarily on unpublished data. Second, Merative Marketscan data were used for many of the model inputs, but data for people aged 65 years or older only included those with supplemental insurance and did not include individuals covered only by Medicare who may face different costs. Third, in the phase 1 analysis, hospitalization data did not differentiate between those in the hospital with incidental COVID-19 vs those in the hospital because of COVID-19 illness, which may have caused some bias in the conditional probabilities of critical illness. This analysis was updated in phase 2 using current COVID-NET data. Fourth, the phase 1 analysis did not account for timing of vaccination and seasonality of COVID-19 illness, but these were incorporated in phase 2 (and later analyses). As a consequence, the phase 1 analysis should be viewed as using a conservative approach to VE. Fifth, symptomatic illness is broadly defined in the HEROES-RECOVER dataset and may include a wide variety of symptom severity. Sixth, several limitations also arose from the scarcity of available data on long COVID. The probability of long COVID included in the model did not reflect the possible association between the probability or duration of long COVID and age and severity of illness. Future phases of the model should incorporate these adjustments, along with current probabilities of long COVID, as more data become available. Cost estimates for long COVID were derived from 2020 to 2021 data and may not reflect current practice patterns or rates of health care use. Overall, these limitations may underestimate the 5-month costs associated with an episode of long COVID. Seventh, an important consideration is the potential effects of vaccination on reducing population transmission of COVID-19 and SARS-CoV-2 infection, which was not included in this analysis. Unfortunately, there are no randomized trials showing vaccination to reduce transmission, and observational studies are subject to many biases. However, if vaccination also reduced transmission, it may have led to higher benefits, increasing the value of vaccination. On the other hand, if prior vaccination were to substantially lower population rates of infection, the value of additional vaccination might worsen (Table 3). Finally, the analysis assumed that the bivalent mRNA (booster) VE against prior strains of COVID-19 would be an appropriate approximation for the new monovalent 2023-2024 COVID-19 mRNA VE against 2023-2024 circulating strains of COVID-19, as VE estimates for the 2023-2024 monovalent vaccine were not yet available.

## Conclusions

This decision analytic modeling study shows that vaccination with an updated 2023 to 2024 COVID-19 mRNA vaccine for all adults in the US may be attractive not only for its expected health gains but also for its economic benefits. As the evidence base for COVID-19 vaccination and burden of illness rapidly evolve, it will be important to continue to update and revise the economic evaluation of vaccination against COVID-19. In particular, higher-quality data are needed to assess vaccine safety and effectiveness and to estimate the burden of disease for pediatric and adolescent age groups. Understanding the health effects and costs of long COVID also requires further investigation. The National Academies of Sciences, Engineering, and Medicine has recently released a report providing a standard definition of long COVID, and this is anticipated to support and help guide future research in this area.^32^ As hospitalizations and deaths due to COVID-19 continue to decline, the burden of long COVID will have increasing importance in determining the economic attractiveness of future updated COVID-19 vaccines.

## Notes

Supplement 1. **eTable 1.** Complete List of Parameters: Probabilities for COVID-19–Related Events **eTable 2.** Complete List of Parameters: Probabilities for Vaccination-Related Events **eTable 3.** Complete List of Parameters: Costs **eTable 4.** Complete List of Parameters: Quality-of-Life Adjustments **eTable 5.** Total Projected Costs and QALYs, Incremental Costs and QALYs, Incremental Cost-Effectiveness Ratios ($/QALY Gained) Per 100 000 (Health Care Sector Perspective) **eTable 6.** Impact Inventory **eTable 7.** Disaggregated COVID-19 Outcomes With and Without 2023-2024 COVID-19 Vaccination, Per 100 000 **eTable 8.** One-Way Sensitivity Analysis, $/QALY Gained, Societal Perspective **eTable 9.** Scenario Analyses: Vaccine Effectiveness, $/QALY Gained, Societal Perspective **eTable 10.** Scenario Analyses: Annual Probability of Symptomatic Illness, $/QALY Gained, Societal Perspective **eTable 11.** Scenario Analyses: Vaccination Setting, $/QALY Gained, Societal Perspective **eTable 12.** Scenario Analyses: Post–COVID-19 Condition Excluded, $/QALY Gained, Societal Perspective **eTable 13.** Scenario Analyses: Lower VE Against Symptomatic COVID Illness, $/QALY Gained, Societal Perspective **eTable 14.** Scenario Analyses: Unrelated Health Care Costs Included, $/QALY Gained, Societal Perspective **eTable 15.** Scenario Analyses: Unrelated Health Care Costs Included, $/QALY Gained, Health Care Sector Perspective **eFigure 1.** Vaccine Effectiveness, >18 Years **eFigure 2.** One-Way Sensitivity Analysis of Most Influential Parameters, Vaccination With 2023-2024 Vaccination Compared With No 2023-2024 Vaccination
eReferences.

Supplement 2. **eTable 1.** Alternate Seasonality-Adjusted Vaccine Inputs by Disease Outcome, Age Group, and Vaccine Dose **eTable 2.** Disaggregated Disease Outcomes and Outcomes Averted for 1-Dose and 2-Dose Strategies, Per 100 000, Phase 2 Estimates **eTable 3.** Scenario Analyses, ICER ($/QALY Gained) **eTable 4.** Cost Per Cases Averted (Societal Perspective) and Number Needed to Vaccinate for 1-Dose and 2-Dose Strategies by Age Group and Type of Disease Outcome **eFigure 1.** Model Schematic **eFigure 2.** Derivation of Seasonality-Adjusted Vaccine Impact **eFigure 3.** Scenario Analysis: Vaccine Dose Cost
eReferences.

Supplement 3.
Data Sharing Statement
