# Supplementary material for: Cost-Effectiveness of 2023-2024 COVID-19 Vaccination in US Adults
Source: JAMA Netw Open. 2025 Aug 7;8(8):e2523688. doi: 10.1001/jamanetworkopen.2025.23688 (PMC12332627; doi:10.1001/jamanetworkopen.2025.23688)
Supplement: Supplement 1. — eTable 1. Complete List of Parameters: Probabilities for COVID-19–Related Events eTable 2. Complete List of Parameters: Probabilities for Vaccination-Related Events eTable 3. Complete List of Parameters: Costs eTable 4. Complete List of Parameters: Quality-of-Life Adjustments eTable 5. Total Projected Costs and QALYs, Incremental Costs and QALYs, Incremental Cost-Effectiveness Ratios ($/QALY Gained) Per 100 000 (Health Care Sector Perspective) eTable 6. Impact Inventory eTable 7. Disaggregated COVID-19 Outcomes With and Without 2023-2024 COVID-19 Vaccination, Per 100 000 eTable 8. One-Way Sensitivity Analysis, $/QALY Gained, Societal Perspective eTable 9. Scenario Analyses: Vaccine Effectiveness, $/QALY Gained, Societal Perspective eTable 10. Scenario Analyses: Annual Probability of Symptomatic Illness, $/QALY Gained, Societal Perspective eTable 11. Scenario Analyses: Vaccination Setting, $/QALY Gained, Societal Perspective eTable 12. Scenario Analyses: Post–COVID-19 Condition Excluded, $/QALY Gained, Societal Perspective eTable 13. Scenario Analyses: Lower VE Against Symptomatic COVID Illness, $/QALY Gained, Societal Perspective eTable 14. Scenario Analyses: Unrelated Health Care Costs Included, $/QALY Gained, Societal Perspective eTable 15. Scenario Analyses: Unrelated Health Care Costs Included, $/QALY Gained, Health Care Sector Perspective eFigure 1. Vaccine Effectiveness, >18 Years eFigure 2. One-Way Sensitivity Analysis of Most Influential Parameters, Vaccination With 2023-2024 Vaccination Compared With No 2023-2024 Vaccination eReferences. [file jamanetwopen-e2523688-s001.pdf]

Supplemental Online Content

Prosser LA, Wallace M, Rose AM, et al. Cost-effectiveness of 2023-2024 COVID-19 vaccination in US adults. *JAMA Netw Open*. 2025;8(8):e2523688. doi:10.1001/jamanetworkopen.2025.23688

eTable 1. Complete List of Parameters: Probabilities for COVID-19–Related Events

eTable 2. Complete List of Parameters: Probabilities for Vaccination-Related Events

eTable 3. Complete List of Parameters: Costs

eTable 4. Complete List of Parameters: Quality-of-Life Adjustments

eTable 5. Total Projected Costs and QALYs, Incremental Costs and QALYs, Incremental Cost-Effectiveness Ratios ($/QALY Gained) Per 100 000 (Health Care Sector Perspective)

eTable 6. Impact Inventory

eTable 7. Disaggregated COVID-19 Outcomes With and Without 2023-2024 COVID-19 Vaccination, Per 100 000

eTable 8. One-Way Sensitivity Analysis, $/QALY Gained, Societal Perspective

eTable 9. Scenario Analyses: Vaccine Effectiveness, $/QALY Gained, Societal Perspective

eTable 10. Scenario Analyses: Annual Probability of Symptomatic Illness, $/QALY Gained, Societal Perspective

eTable 11. Scenario Analyses: Vaccination Setting, $/QALY Gained, Societal Perspective

eTable 12. Scenario Analyses: Post–COVID Condition Excluded, $/QALY Gained, Societal Perspective

eTable 13. Scenario Analyses: Lower VE Against Symptomatic COVID Illness, $/QALY Gained, Societal Perspective

eTable 14. Scenario Analyses: Unrelated Health Care Costs Included, $/QALY Gained, Societal Perspective

eTable 15. Scenario Analyses: Unrelated Health Care Costs Included, $/QALY Gained, Health Care Sector Perspective

eFigure 1. Vaccine Effectiveness, >18 Years

eFigure 2. One-Way Sensitivity Analysis of Most Influential Parameters, Vaccination With 2023-2024 Vaccination Compared With No 2023-2024 Vaccination

eReferences

This supplemental material has been provided by the authors to give readers additional information about their work.

© 2025 Prosser LA et al. *JAMA Network Open*.

Supplemental Materials, Phase 1

eTable 1. Complete List of Input Parameters: Probabilities for COVID-19–Related Events

Age	Base Case	Range for Sensitivity Analysis		Source
		Low	High	
**Annual probability of symptomatic COVID-19[a]**				
18-49 y	0.3145	0.2858	0.3444	[b]
50-64 y	0.2841	0.2438	0.3274	
≥65 y	0.3339	0.2312	0.4510	
**Probability of outpatient visit given symptomatic COVID-19[c]**				
18-49 y	0.157	0.1483	0.1664	[b], [d]
50-64 y	0.215	0.1991	0.2335	
≥65 y	0.244	0.1996	0.3088	
**Probability of emergency department visit given symptomatic illness**				
18-49 y	0.0184	0.0172	0.0196	[b, d]
50-64 y	0.0191	0.0175	0.0210	
≥65 y	0.0394	0.0318	0.0505	
**Probability of Long COVID given outpatient visit or hospitalization[e]**				
18-49 y	0.072	0.058	0.091	[1]
50-64 y	0.072	0.058	0.091	
≥65 y	0.072	0.058	0.091	
**Annual probability of hospitalization**				
18-49 y	0.00144	0.00080	0.00204	[2]
50-64 y	0.00335	0.00216	0.00479	
≥65 y	0.01453	0.00967	0.02090	
**Probability of ICU admission given hospitalization**				
18-49 y	0.123	0.119	0.145	[2]
50-64 y	0.200	0.178	0.208	
≥65 y	0.144	0.138	0.163	
**Probability of ventilator assistance given ICU admission[f]**				
18-49 y	0.525	0.472	0.577	[2]
50-64 y	0.488	0.445	0.532	
≥65 y	0.386	0.342	0.432	
**Probability of long-term sequelae given ICU admission (no ventilator assistance)[g]**				

[a] Using data from December 2022-May 2023.

[b] Unpublished data from HEROS-RECOVER, email communication with Ryan Wiegand, PhD, May 2023.

[c] Conditional probability of outpatient or ED visit was derived using data from Merative™ Marketscan® for the period May 2022-October 2022 combined with symptomatic illness data from HEROS-RECOVER.

[d] Unpublished data from Merative™ Marketscan®, email communication with Fangjun Zhou, PhD, August 2023.

[e] Average prevalence of HEENT, constitutional, pulmonary, musculoskeletal, cognitive, and fatigue symptoms.

[f] Adjusted for ventilator assistance cases not in the ICU.

[g] Assumption: Individuals only develop long-term sequelae after ICU admission.

© 2025 Prosser LA et al. *JAMA Network Open*.

≥18 y	0.004	0.002	0.008	3-8
**Probability of long-term sequelae given ICU admission (with ventilator assistance)**				
18+ y	0.009	0.005	0.018	3-8
**Probability of death given hospitalization (no ICU)**				
18-49 y	0.006	0.002	0.008	2
50-64 y	0.009	0.007	0.016	
≥65 y	0.030	0.022	0.035	
**Probability of death given ICU admission (no ventilator assistance)**				
18-49 y	0.024	0.003	0.040	2
50-64 y	0.047	0.026	0.077	
≥65 y	0.166	0.144	0.233	
**Probability of death given ICU admission (with ventilator assistance)**				
18-49 y	0.284	0.213	0.368	2
50-64 y	0.379	0.301	0.435	
≥65 y	0.628	0.476	0.637	

© 2025 Prosser LA et al. *JAMA Network Open*.

eTable 2. Complete List of Input Parameters: Probabilities for Vaccination-Related Events

Age	Base Case	Range for Sensitivity Analysis		Source
		Low	High	
**Vaccine effectiveness against non-hospitalized symptomatic illness[h]**				
>18 y	0.269	0.088	0.418	[9,10]
**Vaccine effectiveness against hospitalization[i]**				
>18 y	0.269	0.088	0.418	[9,10]
**Vaccine effectiveness against hospitalization in ICU[j]**				
>18 y	0.403	0.191	0.671	[10]
**Vaccine effectiveness against death[k]**				
>18 y	0.403	0.191	0.671	[10]
**Vaccine adverse event: Probability of systemic reaction[l]**				
18-49 y	0.106	0.073	0.148	[11,12]
50-64 y	0.106	0.073	0.148	
>65 y	0.137	0.107	0.171	
**Vaccine adverse event: Probability of anaphylaxis**				
Anaphylaxis	0.00000495	0.0000032	0.0000074	[13]
Death given anaphylaxis	0	0	0.00966	Assumption, [14]
**Vaccine adverse event: Probability of myocarditis/pericarditis**				
18 - 29 y[m]	0.0000238	0.0000085	0.0000838	[15]
30-39 y	0.0000087	0.0000008	0.0000375	[15]
40-49 y	0	0	0	Assumption
50-64 y	0	0	0	Assumption
>65 y	0	0	0	Assumption
Probability of death given myocarditis	0.0005	0	0.001	Expert opinion

[h] Using data on bivalent booster data from September 2022-May 2023. Range includes minimum and maximum from both data sources, minimum: conservative (VE at 180 days=0) and maximum: optimistic approaches (VE at 180 days=VE at 365 days); assumed same as hospitalization.

[i] Bivalent booster data from Sep 2022-May 2023. Range includes minimum and maximum from both data sources, applying min: conservative (VE at 180 days=0) and max: optimistic approaches (VE at 180 days=VE at 365 days).

[j] Bivalent booster data from Sep 2022-May 2023.

[k] Bivalent booster data from Sep 2022-May 2023.

[l] Moderate/severe systemic reactions; ranges are derived.

[m] Used to calculate a weighted average for the 18-49 year age group in the model: 0.00001083 (Range: 0.00000310- 0.00004043).

© 2025 Prosser LA et al. *JAMA Network Open*.

eTable 3. Complete List of Input Parameters: Costs

Age	Base Case	Range for Sensitivity Analysis		Source
		Low	High	
**Vaccine cost**[n]				
All manufacturers	$120	$30	$200	[16]
**Vaccine administration**				
Per dose[o]	$20.33	$18.07	$26.58	[17]
Recipient time by setting (hr)				
Mass vaccination	0.195	0	0.390	[18]
Physician office	1.19	0.17	2	[18]
Pharmacy	0.25	0.083	0.5	[18], expert opinion
Proportion by setting				
Mass vaccination[p]	0.10	0.075	0.155	[19]
Physician office visit[q]	0.256	0.221	0.294	[19]
Pharmacy[r]	0.644	0.625	0.663	[19]
**Systemic reaction**[s]				
Physician visit	$90.82	$82.72	$115.84	[17]
Patient time for physician visit	-	-	-	
Productivity loss (days)	1 d	-	-	Assumption
**Anaphylaxis**[t]				
Hospitalization	$5035			[20]
Productivity loss (days)	1	1	3	[21]
**Myocarditis**[u]				
Hospitalization	$75,927			[22]
Productivity loss (days)	4	0	14	[23,24], Expert opinion

[n] Lower bound reflects price of bivalent boosters as of March 2023.

[o] CPT 90471.

[p] Includes vaccinations given at mass vaccination centers, community centers, work, and school May-June 2023 02/10/23 - 05/01/23.

[q] Includes vaccinations given at health dept, other clinics, hospitals, and physician offices NIS-CCM timeframe: May-June 2023 Ipsos/NORC timeframe: 02/10/23 - 05/01/23.

[r] Includes all vaccinations given at stores NIS-CCM timeframe: May-June 2023 Ipsos/NORC timeframe: 02/10/23 - 05/01/23.

[s] CPT 99213 (20-29 min E&M visit) Assumption, included in 1 day productivity loss.

[t] HCUP-NIS 2012 estimates (mean LOS = 4.9 days) adjusted to 1 days LOS and inflated to 2023US$.

[u] HCUP-NIS 2014 estimates (mean LOS = 7.4 days) adjusted to 4 days LOS and inflated to 2023US$.

© 2025 Prosser LA et al. *JAMA Network Open*.

**Testing**[v]				
18+ y	$8	$8	$62	[25,26]
Probability of testing	0.05	0.02	0.20	[27] , Assumption
Recipient time (hours)	0.50	0.25	1.50	Assumption
**Over the counter medication cost**				
18-49 y	$4.12	0	0	Assumption
50-64 y	$4.12	0	0	Assumption
≥65 y	$4.12	0	0	Assumption
**Nirmatrelvir-r**				
Probability| Outpatient visit				
18-49 y	0.1751	0.10	0.30	[w],[x], Assumption
50-64 y	0.2696	0.10	0.50	
≥65 y	0.2739	0.10	0.50	
Cost[y]	$530	$0	$1200	[28]
**Outpatient visit**				
Direct medical				
18-49 y	$372	$370	$375	[x]
50-64 y	$380	$377	$384	
≥65 y	$391	$386	$396	
Patient time (hours)[z]	1.08	0.54	1.62	[29,30]
Duration of illness (days)	4.6	3.6	5.7	[31]
**Emergency department**				
Direct medical				
18-49 y	$582	$540	$1,312	[32]
50-64 y	$582	$540	$1,312	
≥65 y	$582	$540	$1,312	
Patient time (hours)	6.4	2.80	14.6	[33]
**Long COVID**[aa]				

[v] Base case is average cost of 1 at home test. Upper range based on KFF analysis for PCR testing median costs.

[w] Unpublished data HealthVerity, email communication with Melisa Shah, MD, August 2023.

[x] Unpublished data from Merative™ Marketscan®, email communication with Fangjun Zhou, PhD, August 2023.

[y] Government price.

[z] Estimated assuming 50% of COVID-19 outpatient visits are in person and 50% are virtual.

[aa] Adjusted to US$2023 using a blended 2020/2021 adjustor. Estimates reflect proportion of those with Long COVID who have cognitive symptoms/fatigue.

© 2025 Prosser LA et al. *JAMA Network Open*.

Outpatient visits, direct medical	$1091	$1018	$1165	[34, bb]
Duration of illness (days)	150	-	-	Assumption
Proportion of patients with productivity loss	0.51	-	-	[1]
Productivity loss due to Long COVID	0.50	-	-	Assumption
**Hospitalization**[cc]				
Direct medical				
18-49 y	$32,514	$28,505	$36,523	[x]
50-64 y	$32.854	$31,450	$34,258	
≥65 y	$20,648	$20,295	$21,000	
Length of stay				
18-49 y	9.0	8.4	9.8	[x]
50-64 y	10.6	10.2	10.8	
≥65 y	12.0	11.8	12.2	
**ICU stay (no ventilator assistance)** [cc]				
Direct medical				
18-49 y	$37,159	$30,116	$44,203	[x]
50-64 y	$46,727	$40,269	$53,186	
≥65 y	$23,220	$22,408	$24,032	
Length of stay				
18-49 y	10.6	9.4	11.8	[x]
50-64 y	15.6	14.4	16.8	
≥65 y	15.4	14.8	16.2	
**ICU stay (with ventilator assistance)** [cc]				
Direct medical				
18-49 y	$245,432	$168,362	$322,503	[x]
50-64 y	$169,189	$140,250	$198,129	
≥65 y	$55,257	$50,705	$59,809	
Duration of illness (days)				
18-49 y	48.2	37.4	59.2	[x]
50-64 y	40.6	35.6	45.4	
≥65 y	28.8	26.0	31.6	
Post discharge care	$10,531.44	$8,425.15	$12,637.72	[35,36]
**ARDS follow up care, direct medical**				
≥18 y	$23,561	$9,790	$53,337	[37]

[bb] 5-month costs derived from 3-month and 6-month costs using linear interpolation.

[cc] Productivity loss=2x length of stay.

© 2025 Prosser LA et al. *JAMA Network Open*.

**Long term sequelae from ICU treated illness (lifetime discounted costs), direct medical**				
18-49 y	$652,521	$326,260	$1,305,041	38-41
50-64 y	$438,325	$219,163	$876,650	
≥65 y	$136,744	$68,372	$273,489	
**Daily productivity**				
18-49 y	$ 110.50	-	-	42
50-64 y	$ 124.50	-	-	
≥65 y	$38.50	-	-	
**Mean hourly earnings**	$33.74	$21.31	$50.05	43
**Lifetime productivity losses due to death[dd]**				
18-49 y	$1,191,174	$1,191,174	$2,382,347	42
50-64 y	$653,522	$653,522	$1,307,044	
≥65 y	$184,320	$184,320	$368,640	
**Unrelated health care costs (lifetime, discounted)**				
18-49 y	$221,439	-	-	44
50-64 y	$225,392	-	-	
≥65 y	$120,509	-	-	

[dd] Discounted lifetime productivity loss was derived using estimates published by Gross et al. (2019) by age and adjusted to 2023$.

© 2025 Prosser LA et al. *JAMA Network Open*.

eTable 4. Complete List of Input Parameters: Quality-of-Life Adjustments

Age	Base Case	Range for Sensitivity Analysis		Source
		Low	High	
**Illness (QALY loss per episode)[ee]**				
Symptomatic	0.006	0.004	0.008	[ff]
Hospitalization	0.027	-		
Critical Illness	0.054	-	-	
Long covid[gg]	0.067	0.038	0.088	
**Systemic reaction (QALY loss)[hh]**				
18-49 y	0.0004	0.0003	0.0005	Assumption
50-64 y	0.0004	0.0003	0.0005	Assumption
≥65 y	0.0004	0.0003	0.0005	Assumption
**Anaphylaxis (QALY loss)**				
≥18 y	0.0137	0.0135	0.0139	[45]
**Myocarditis/pericarditis (QALY loss)[ii]**				
Acute illness	0.010	0.0086	0.0112	[46]
**Long term complications from ICU illness (utility)[jj]**	0.730	0.680	0.790	[47]
**COVID-19 spillover (QALY loss)[kk]**				
Symptomatic				
18-64 y	0.002	0.001	0.003	[48]
≥65 y	0.004	0	0.023	
Hospitalization				
18-64 y	0.006	0.004	0.009	[48]
≥65 y	0.007	0	0.026	
Critical Illness (with ventilator assistance) (QALY loss)				
18-64 y	0.012	0	0.028	[48]
≥65 y	0.019	0	0.050	
**Long COVID[ll]**				
18-64 y	0.015	0.000	0.067	[48]
≥65 y	0.019	0.000	0.076	

[ee] QALY loss per person. Derived using the ratio of ICU to hospitalization QALY loss (2x) applied to the EQ5D hospitalization QALY loss. (48-50)

[ff] Unpublished data from SARS-CoV-2 Epidemiology And Response in Children (SEARCh), Prospective Assessment of COVID-19 in a Community (PACC), and Coronavirus Household Evaluation and Respiratory Testing (C-HEaRT) studies, email communication with Sheroi Johnson, MPH, May 2023.

[gg] QALY loss adjusted to 5-month duration.

[hh] Assumption, QALY loss equal to QALY loss associated with one day of COVID-19 illness, EQ-5D survey.

[ii] Assumption of 2 weeks duration of illness for covid-related myocarditis.

[jj] Derived from health utility weight for pulmonary fibrosis.

[kk] Adjusted using the ratio of 2023 survey to 2021 survey estimates to reflect updated preferences for COVID QOL.

[ll] QALY loss adjusted to 5-month duration.

© 2025 Prosser LA et al. *JAMA Network Open*.

**Discounted QALEs[mm]**				
18-49 y	20.21			49,50
50-64 y	12.28			
≥65 y	6.53			

ICU = intensive care unit ARDS = acute respiratory distress syndrome; QALY = quality-adjusted life-year; QALE = quality-adjusted life expectancy; Spillover QALY loss refers to QALY loss experienced by a caregiver

[mm] Discounted QALE was approximated using life expectancy from US life tables for the mid-point age in each age group, age-adjusted health utility using published weights for the US adult population, and an annual 3% discount rate. Note: Although individuals who die due to COVID-19 related complications may have below-average remaining life expectancy, this analysis does not include any adjustments for underlying health status and uses the average health utility and life expectancy by age.

© 2025 Prosser LA et al. *JAMA Network Open*.

eTable 5. Total Projected Costs and QALYs, Incremental Costs and QALYs, Incremental Cost-Effectiveness Ratios ($/QALY Gained) Per 100 000 (Health Care Sector Perspective)

Age	Strategy	Projected costs	Incremental Costs	Projected QALYs	Incremental QALYs	$/QALY gained
18-49 y	No 2023-2024 COVID-19 vaccination	$10,201,398	NA	2020707	NA	NA
	Vaccination (2023-2024 COVID-19 vaccine)	$21,410,058	$11,208,660	2020794	87.5	$128,063
50-64 y	No updated 2023-2024 COVID-19 vaccination	$20,976,166	NA	1227583	NA	NA
	Vaccination (2023-2024 COVID-19 vaccine)	$28,776,903	$7,800,737	1227721	137.7	$56,668
≥65 y	No updated 2023-2024 COVID-19 vaccination	$40,204,438	NA	651995	NA	NA
	Vaccination (2023-2024 COVID-19 vaccine)	$43,137,813	$2,933,375	652355	360.5	$8,138

© 2025 Prosser LA et al. *JAMA Network Open*.

eTable 6. Impact Inventory

Type of impact	Healthcare sector perspective	Societal perspective	Comments
**Formal healthcare sector**			
Health outcomes			
Longevity effects	☑	☑	Vaccination helps prevent COVID-19 and related complications, including death
Health related quality of life effects	☑	☑	Health states included COVID-19 related illness, related complications, and vaccination-related adverse events
Other health effects	☑	☑	Cases of COVID-19, hospitalizations, deaths, and adverse events included
Medical costs			
Medical costs paid by third-party payers	☑	☑	Included costs of provider visits, hospitalizations, etc.
Medical costs paid for out-of-pocket	☑	☑	Over-the-counter medications
Future related medical costs	☑	☑	Long-term sequelae costs due to COVID-related hospitalization or vaccination-related adverse events
Future unrelated medical costs	☐	☐	Not included
**Informal healthcare sector**			
Patient time costs	☐	☑	Included as part of vaccination cost
Unpaid caregiver costs	☐	☐	Not included
Transportation costs	☐	☐	Not included
**Non-healthcare sectors**			
Productivity	☐	☑	Included with cases of COVID-19 and related complications, and averted deaths
Consumption	☐	☑	
Education	☐	☐	Not included
Cost of crimes related to intervention	☐	☐	Not applicable to condition

© 2025 Prosser LA et al. *JAMA Network Open*.

eTable 7. Disaggregated COVID-19 Outcomes With and Without 2023-2024 COVID-19 Vaccination, Per 100 000

		Cases				Cases Averted			
**Age**	**Strategy**	**Cases**	**Hospitalizations**	**ICU**	**Deaths**	**Cases**	**Hospitalizations**	**ICU**	**Deaths**
18-49 y	No 2023-2024 COVID-19 vaccination	31,450	144	17.7	3.6	-	-	-	-
	Vaccination (2023-2024 COVID-19 vaccine)	22,990	105	10.6	2.2	8,460	39	7.1	1.4
50-64 y	No 2023-2024 COVID-19 vaccination	28,410	335	67.1	16.4	-	-	-	-
	Vaccination (2023-2024 COVID-19 vaccine)	20,768	245	40.0	9.9	7,642	90	27.0	6.6
≥65 y	No 2023-2024 COVID-19 vaccination	33,390	1453	209.3	109.4	-	-	-	-
	Vaccination (2023-2024 COVID-19 vaccine)	24,408	1062	124.9	66.0	8,982	391	84.3	43.4

© 2025 Prosser LA et al. *JAMA Network Open*.

eTable 8. One-Way Sensitivity Analysis, $/QALY Gained, Societal Perspective

Variable Description	Variable Low	Variable Base	Variable High	ICER ($/QALY gained)	
				With variable lower bound	With variable upper bound
**18-49 y**					
Cost, vaccine dose	30	120	200	$12,760	$206,990
VE, symptomatic COVID-19	0.088	0.269	0.418	$239,912	$74,113
VE, critical illness (ICU, death)	0.191	0.403	0.671	$159,023	$77,868
Probability, hospitalization	0.000803677	0.001439	0.002037921	$160,406	$84,396
QALYs lost, symptomatic COVID-19	0.004	0.006	0.008	$143,282	$96,865
Probability, death given ICU with ventilator	0.213	0.284	0.368	$126,863	$103,910
VE, hospitalization	0.088	0.269	0.418	$127,559	$105,795
Lifetime productivity cost	1191174	1191174	2382347	$115,588	$96,019
Time (h), vaccination at doctor's office	0.17	1.19	2	$105,522	$123,581
Probability, symptomatic COVID-19	0.2858	0.3145	0.3444	$124,619	$107,252
**50-64 y**					
Cost, vaccine dose	30	120	200	CS	$83,902
VE, critical illness (ICU, death)	0.191	0.403	0.671	$73,332	CS
Probability, hospitalization	0.002157669	0.003354	0.004788498	$65,481	CS
VE, symptomatic COVID-19	0.088	0.269	0.418	$54,411	$11,562
Lifetime productivity cost	653522	653522	1307044	$25,787	CS
VE, hospitalization	0.088	0.269	0.418	$42,256	$12,604
Probability, death given ICU with ventilator	0.301	0.379	0.435	$33,461	$21,089
Time (h), vaccination at doctor's office	0.17	1.19	2	$19,387	$30,869
Probability, ICU given hospitalization	0.178	0.2	0.208	$32,237	$23,608
Probability, ventilator given ICU	0.445	0.488	0.532	$29,904	$21,858
**≥65 y**					
Cost, vaccine dose	30	120	200	CS	$10,090
Probability, hospitalization	0.009672913	0.014533	0.020898535	CS	$6,352
VE, hospitalization	0.088	0.269	0.418	$3,910	CS
VE, critical illness (ICU, death)	0.191	0.403	0.671	$2,909	CS
Lifetime productivity cost	184320	184320	368640	CS	CS
VE, symptomatic COVID-19	0.088	0.269	0.418	CS	CS
Time (h), vaccination at doctor's office	0.17	1.19	2	CS	CS
Time (h), vaccination at pharmacy	0.083	0.25	0.5	CS	CS
Cost, vaccine administration	18.07	20.23	26.58	CS	CS
Probability, symptomatic COVID-19	0.1996	0.244	0.3088	CS	CS

QALY = quality-adjusted life year; VE = vaccine effectiveness; h= hours; ICU = intensive care unit; ICER = incremental cost-effectiveness ratio; CS = cost savings

© 2025 Prosser LA et al. *JAMA Network Open*.

eTable 9. Scenario Analyses: Vaccine Effectiveness, $/QALY Gained, Societal Perspective

	Base Case	Scenario 1 All lower bound	Scenario 2 All upper bound	Scenario 3 VE against hospitalization applied to all endpoints	Scenario 4 VE against critical illness applied to all endpoints
Vaccine effectiveness inputs					
Symptomatic illness	0.269	0.088	0.418	0.269	0.403
Hospitalization, uncomplicated	0.269	0.088	0.418	0.269	0.403
Hospitalization, critical illness	0.403	0.191	0.671	0.269	0.403
Death	0.403	0.191	0.671	0.269	0.403
$/QALY gained					
18-49 y	$115,588	$435,886	$45,376	$141,155	$70,928
50-64 y	$25,787	$199,830	CS	$51,792	$3,001
≥65 y	CS	$51,782	CS	CS	CS

CS = cost savings

© 2025 Prosser LA et al. *JAMA Network Open*.

eTable 10: Scenario Analyses: Annual Probability of Symptomatic Illness, $/QALY Gained, Societal Perspective

Age Group	Base Case	0.1	0.2	0.3	0.4	0.5
18-49 y	$115,588	$229,724	$160,000	$120,013	$94,082	$75,905
50-64 y	$25,787	$48,937	$34,724	$24,324	$16,384	$10,123
≥65 y	CS	CS	CS	CS	CS	CS

Note: Base case annual probability of symptomatic COVID-19 is 0.3145 for 18-49 years, 0.2841 for 50-64 years, and 0.3339 for 65+ years.

eTable 11. Scenario Analysis: Vaccination Setting, $/QALY Gained, Societal Perspective

Age Group	Base case	100% Pharmacy	100% physician office	100% mass vaccination
18-49 y	$115,588	$106,523	$142,759	$104,403
50-64 y	$25,787	$20,024	$43,063	$18,676
≥65 y	CS	CS	CS	CS

CS = cost savings

© 2025 Prosser LA et al. *JAMA Network Open*.

eTable 12. Scenario Analysis: Post–COVID-19 Condition Excluded, $/QALY Gained, Societal Perspective

Age Group	Base case	Post–COVID-19 condition excluded
18-49 y	$115,588	$133,402
50-64 y	$25,787	$33,724
≥65 y	CS	CS

CS = cost savings

eTable 13. Scenario Analysis: Lower VE Against Symptomatic COVID Illness, $/QALY Gained, Societal Perspective

Age Group	Base case	Lower VE against symptomatic illness*
18-49 y	$115,588	$239,913
50-64 y	$25,787	$54,411
≥65 y	CS	CS

CS = cost savings

*VE set to lower bound

eTable 14. Scenario Analysis: Unrelated Health Care Costs Included, $/QALY Gained, Societal Perspective

Age Group	Base case	Unrelated health care costs included
18-49 y	$115,588	$119,225
50-64 y	$25,787	$36,524
≥65 y	CS	$2,403

CS = cost savings

eTable 15. Scenario Analysis: Unrelated Health Care Costs Included, $/QALY Gained, Health Care Sector Perspective

Age Group	Base case	Unrelated health care costs included
18-49 y	$128,063	$131,700
50-64 y	$56,668	$67,404
≥65 y	$8,138	$22,645

© 2025 Prosser LA et al. *JAMA Network Open*.

eFigure 1. Vaccine Effectiveness, >18 Years

a. Hospitalization vaccine effectiveness, VISION and IVY[9,10]

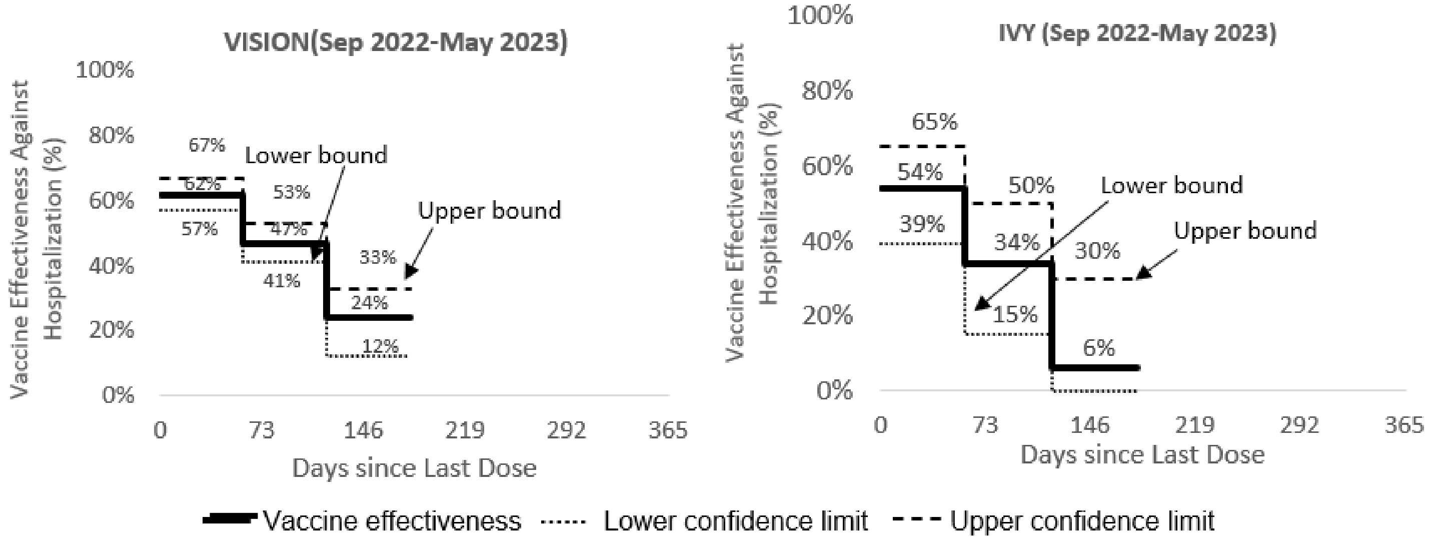

b. Optimistic and conservative scenario assumptions[9,10]

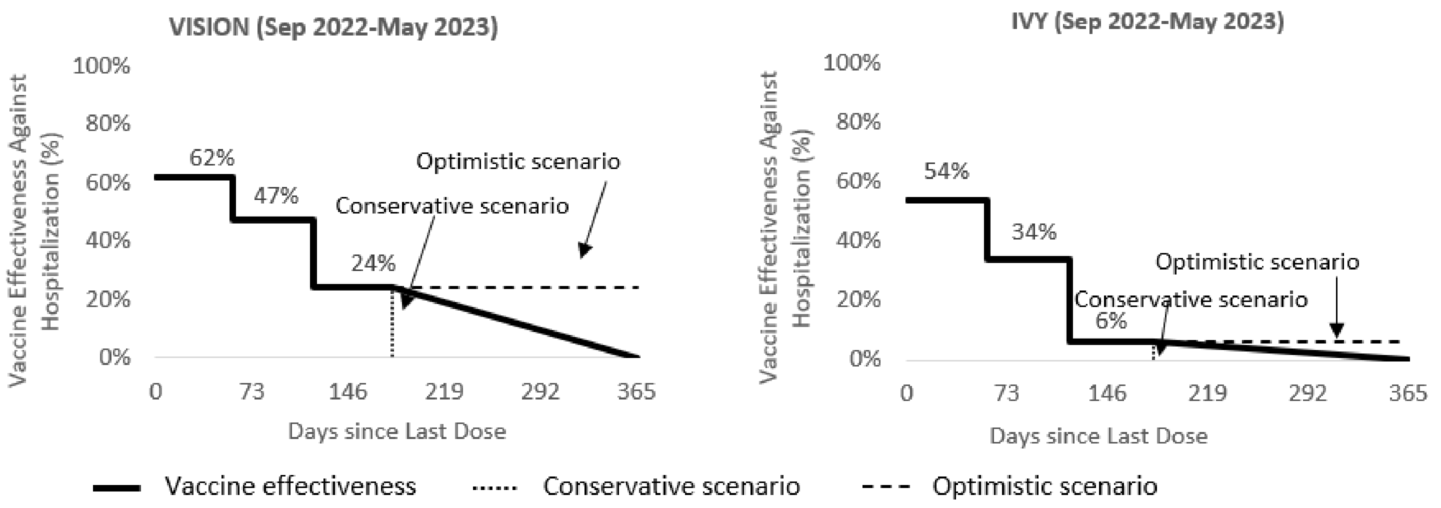

© 2025 Prosser LA et al. *JAMA Network Open*.

c. Vaccine effectiveness against hospitalization[9,10]

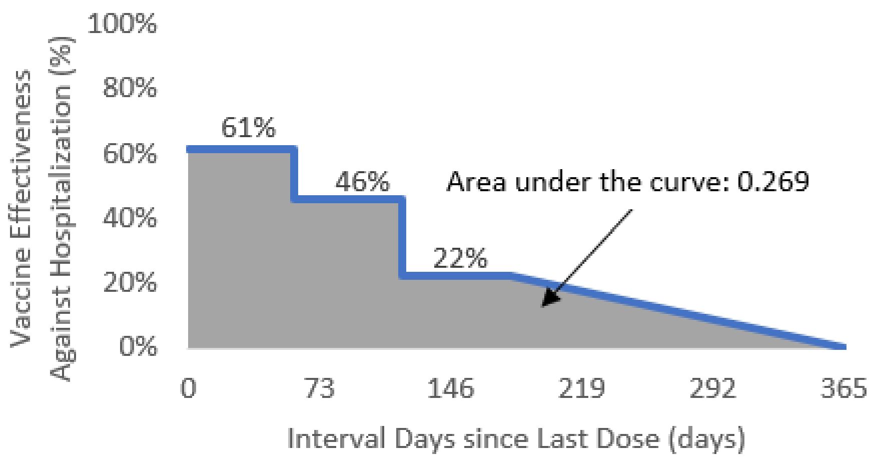

d. Vaccine effectiveness against critical illness (ICU stays and hospitalizations)

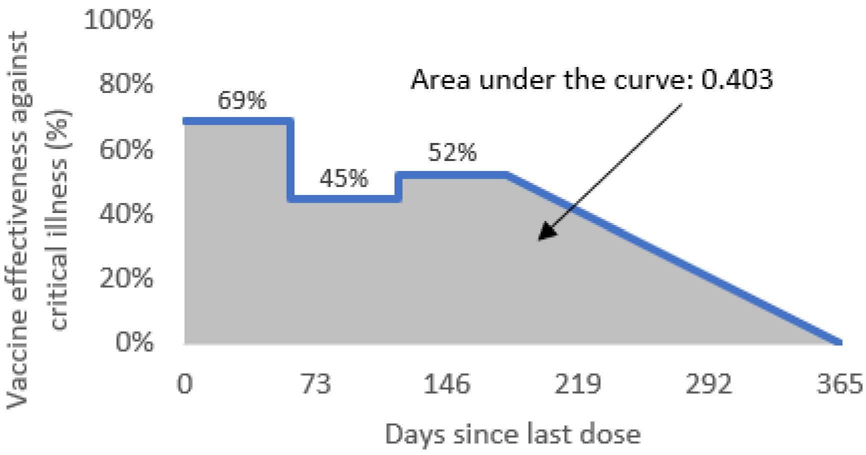

© 2025 Prosser LA et al. *JAMA Network Open*.

eFigure 2. One-Way Sensitivity Analysis of Most Influential Parameters, Vaccination With 2023-2024 Vaccination Compared With No 2023-2024 Vaccination

a. **50-64 y**

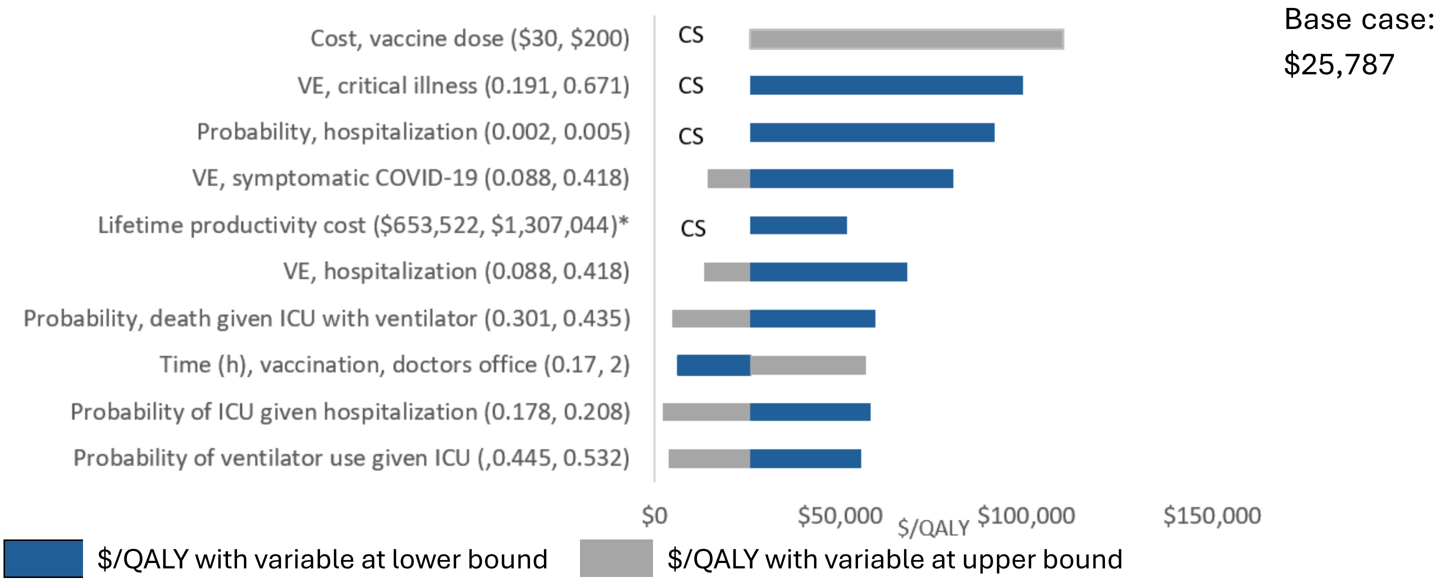

* Lower input is equal to the base case

QALY = Quality adjusted life year; ICU = Intensive care unit; VE = vaccine effectiveness; CS = cost saving

b. **>65 y ***

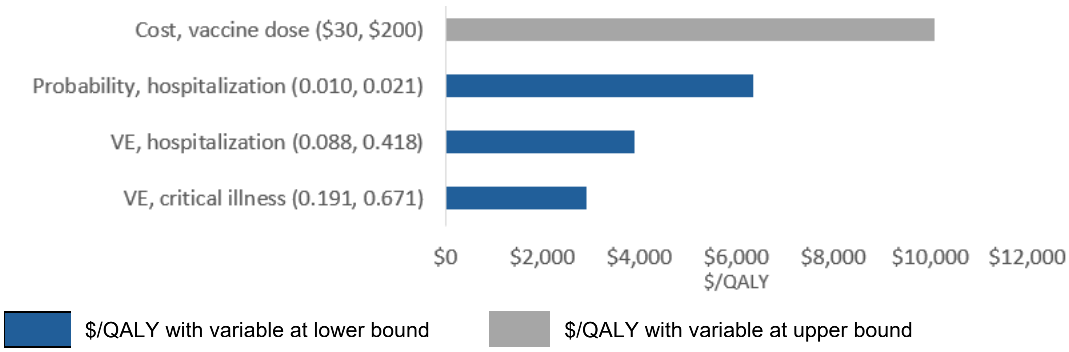

*Base case ICER for vaccination for the 65+ age group is cost saving. The other most influential parameters (lifetime productivity cost; VE, symptomatic illness; time, vaccination, doctor's office; mean hourly wage; time, vaccination, pharmacy; cost, vaccine administration; probability, outpatient visit) were cost saving for both high and low values of parameter input ranges.

QALY = Quality adjusted life year; ICU = Intensive care unit; VE = vaccine effectiveness

© 2025 Prosser LA et al. *JAMA Network Open*.

eReferences

1. Montoy JC, Ford J, Yu H. Prevalence of symptoms ≤12 months after acute illness, by COVID-19 testing status among adults — United States, December 2020–March 2023. MMWR Morb Mortal Wkly Rep 2023;72:859–865. doi:http://dx.doi.org/10.15585/mmwr.mm7232a2
2. COVID-NET, Oct 2022-Mar 2023. https://www.cdc.gov/covid/php/covid-net/index.html
3. Chan L, Chaudhary K, Saha A, et al. AKI in Hospitalized Patients with COVID-19. J Am Soc Nephrol. Jan 2021;32(1):151-160. doi:10.1681/asn.2020050615
4. Gottlieb M, Sansom S, Frankenberger C, Ward E, Hota B. Clinical course and factors associated with hospitalization and critical illness among COVID-19 patients in Chicago, Illinois. Acad Emerg Med. Oct 2020;27(10):963-973. doi:10.1111/acem.14104
5. Hariri LP, Knipe RS. ARDS related to COVID-19 expected to cause increase of pulmonary fibrosis. Massachusetts General Hospital. https://advances.massgeneral.org/research-and-innovation/article.aspx?id=1238
6. Lo LJ, Go AS, Chertow GM, et al. Dialysis-requiring acute renal failure increases the risk of progressive chronic kidney disease. Kidney Int. Oct 2009;76(8):893-9. doi:10.1038/ki.2009.289
7. Merkler AE, Parikh NS, Mir S, et al. Risk of ischemic stroke in patients with Coronavirus Disease 2019 (COVID-19) vs patients with influenza. JAMA Neurol. Jul 2 2020;doi:10.1001/jamaneurol.2020.2730
8. Richardson S, Hirsch JS, Narasimhan M, et al. Presenting characteristics, comorbidities, and outcomes among 5700 patients hospitalized with COVID-19 in the New York City Area. JAMA. May 26 2020;323(20):2052-2059. doi:10.1001/jama.2020.6775
9. Link-Gelles R. Monovalent and bivalent VE against hospitalization among adults aged ≥18 years, IVY Network. U.S. Centers for Disease Control and Prevention. https://www.cdc.gov/acip/downloads/slides-2023-06-21-23/02-COVID-Havers-Galang-Link-Gelles-508.pdf
10. Link-Gelles R. Monovalent and bivalent VE, against hospitalization and critical illness by Omicron subvariant in adults ≥18 years, VISION Network. U.S. Centers for Disease Control and Prevention. https://www.cdc.gov/acip/downloads/slides-2023-06-21-23/02-COVID-Havers-Galang-Link-Gelles-508.pdf
11. Fact sheet for healthcare providers administering vaccine: Emergency use authorization of Moderna COVID-19 Vaccine, Bivalent (Original and Omicron BA.4/BA.5) (2023).
12. Fact sheet for healthcare providers administering vaccine: Emergency use authorization of Pfizer- Biontech COVID-19 vaccine, Bivalent (Original and Omicron BA.4/BA.5) (2023).
13. Klein NP, Lewis N, Goddard K, et al. Surveillance for adverse events after COVID-19 mRNA vaccination. JAMA. 2021;326(14):1390-1399. doi:10.1001/jama.2021.15072
14. Su JR, Moro PL, Ng CS, Lewis PW, Said MA, Cano MV. Anaphylaxis after vaccination reported to the Vaccine Adverse Event Reporting System, 1990-2016. J Allergy Clin Immunol. Apr 2019;143(4):1465-1473. doi:10.1016/j.jaci.2018.12.1003
15. Goddard K, Hanson KE, Ned Lewis. Incidence of myocarditis/pericarditis following mRNA COVID-19 vaccination among children and younger adults in the United States. Annals of Internal Medicine. 2022;175(12):1169-1771. doi:10.7326/m22-2274 %m 36191323
16. Kates J, Cox C, Josh Michaud. How much could COVID-19 vaccines cost the U.S. after commercialization? Kaiser Family Foundation. Accessed September 1, 2023. https://www.kff.org/coronavirus-covid-19/issue-brief/how-much-could-covid-19-vaccines-cost-the-u-s-after-commercialization/
17. Centers for Medicare & Medicaid Service. Physician Fee Schedule. https://www.cms.gov/medicare/physician-fee-schedule/search?Y=0&T=0&HT=0&CT=3&H1=90471&M=5
18. Prosser L, O'Brien M, Molinari N, et al. Non-traditional settings for influenza vaccination of adults: Costs and cost-effectiveness. Pharmacoeconomics. 2008;26(2):163-178. doi:10.2165/00019053-200826020-00006
19. Centers for Disease Control and Prevention. National and state-specific estimates of settings where adults received influenza, updated COVID-19, and RSV vaccinations, 2023-2024 respiratory virus season, United States. Accessed October, 2024. https://www.cdc.gov/vaccines/imz-managers/coverage/national-state-vaccination-estimates.html
20. Candrilli S, Kurosky SK. Recent trends in anaphylaxis-related hospitalization in the United States. Value in Health. 2015;18(7):A503. doi:10.1016/j.jval.2015.09.1428

© 2025 Prosser LA et al. *JAMA Network Open*.

21. Shimabukuro T, Cole M, Su JR. Reports of anaphylaxis after receipt of mRNA COVID-19 vaccines in the US-December 14, 2020-January 18, 2021. Jama. Mar 16 2021;325(11):1101-1102. doi:10.1001/jama.2021.1967
22. Khorolsky C, Shi J, Chkhikvadze T. Trends in hospitalization costs, length of stay and complications among patients with acute myocarditis: A 10-year United States perspective. Journal of the American College of Cardiology. 2019;73(9_Supplement_1):935-935. doi:10.1016/S0735-1097(19)31542-6
23. Marshall M, Ferguson I, Lewis P, et al. Symptomatic acute myocarditis in seven adolescents following Pfizer-BioNTech COVID-19 Vaccination. Pediatrics. 2021;doi:10.1542/peds.2021-052478
24. Shimabukuro T. COVID-19 vaccine safety updates. Accessed November, 2024. https://stacks.cdc.gov/view/cdc/110919
25. Lo J, Cox C, Amin K, Telesford I, Dawson L, Jennifer Kates. Prices for COVID-19 testing. Kaiser Family Foundation. https://www.healthsystemtracker.org/brief/prices-for-covid-19-testing/#Prices%20for%20COVID-19%20tests%20in%20the%20outpatient%20setting,%20among%20people%20with%20large%20employer%20health%20coverage,%202021
26. Walmart. COVID-19 test kits. Accessed September 1, 2023. https://www.walmart.com/browse/home-diagnostic-tests/covid-19-test-kits/976760_1005860_542089_3092061
27. Rader B GA, Iuliano AD,. Use of at-home COVID-19 tests — United States, August 23, 2021–March 12, 2022. MMWR Morb Mortal Wkly Rep 2022. 71:489-494. doi:http://dx.doi.org/10.15585/mmwr.mm7113e1
28. Recht H. Paxlovid has been free so far. Next year, sticker shock awaits. KFF Health News. https://kffhealthnews.org/news/article/paxlovid-covid-sticker-shock-insurance/#:~:text=The%20U.S.%20government%20has%20so,in%20a%20July%20earnings%20call.
29. Khairat S, Pillai M, Edson B, Gianforcaro R. Evaluating the telehealth experience of patients with COVID-19 symptoms: recommendations on best practices. J Patient Exp. Oct 2020;7(5):665-672. doi:10.1177/2374373520952975
30. Ray KN, Chari AV, Engberg J, Bertolet M, Mehrotra A. Opportunity costs of ambulatory medical care in the United States. Am J Manag Care. Aug 2015;21(8):567-74. doi:https://pmc.ncbi.nlm.nih.gov/articles/PMC8085714/
31. Centers for Disease Control and Prevention. Respiratory Virus Transmission Network Work Productivity and Activity Impairment Survey https://www.cdc.gov/flu-vaccines-work/php/vaccine-effectiveness/rtvn-flu.html
32. Fiedler M, Song Z. Estimating potential spending on COVID-19 care. 2020. https://www.brookings.edu/research/estimating-potential-spending-on-covid-19-care/#cancel
33. Rose L, Scales DC, Atzema C, et al. Emergency department length of stay for critical care admissions. A population-based study. Ann Am Thorac Soc. Aug 2016;13(8):1324-32. doi:10.1513/AnnalsATS.201511-773OC
34. Pike J, Kompaniyets L, Lindley MC, Saydah S, Miller G. Direct medical costs associated with post-COVID-19 conditions among privately insured children and adults. Prev Chronic Dis. Feb 9 2023;20:E06. doi:10.5888/pcd20.220292
35. Bartsch SM, Ferguson MC, McKinnell JA, et al. The potential health care costs and resource use associated with COVID-19 in the United States. Health Aff (Millwood). Jun 2020;39(6):927-935. doi:10.1377/hlthaff.2020.00426
36. Braun L, Riedel AA, Cooper LM. Severe sepsis in managed care: analysis of incidence, one-year mortality, and associated costs of care. J Manag Care Pharm. Nov-Dec 2004;10(6):521-30. doi:10.18553/jmcp.2004.10.6.521
37. Ruhl AP, Huang M, Colantuoni E, et al. Healthcare utilization and costs in ARDS survivors: A 1-year longitudinal national US multicenter study. Intensive Care Med. Jul 2017;43(7):980-991. doi:10.1007/s00134-017-4827-8
38. Fan Y, Bender SD, Conoscenti CS, et al. Hospital-based resource use and costs among patients with idiopathic pulmonary fibrosis enrolled in the Idiopathic Pulmonary Fibrosis Prospective Outcomes (IPF-PRO) Registry. Chest. Jun 2020;157(6):1522-1530. doi:10.1016/j.chest.2019.12.041
39. Silver SA, Long J, Zheng Y, Chertow GM. Cost of acute kidney injury in hospitalized patients. J Hosp Med. Feb 2017;12(2):70-76. doi:10.12788/jhm.2683
40. Voigt J, Sasha John M, Taylor A, Krucoff M, Reynolds MR, Michael Gibson C. A reevaluation of the costs of heart failure and its implications for allocation of health resources in the United States. Clin Cardiol. May 2014;37(5):312-21. doi:10.1002/clc.22260

© 2025 Prosser LA et al. *JAMA Network Open*.

41. Saran R, Robinson B, Abbott KC, et al. US Renal Data System 2019 annual data report: Epidemiology of kidney disease in the United States. Am J Kidney Dis. Jan 2020;75(1 Suppl 1):A6-A7. doi:10.1053/j.ajkd.2019.09.003
42. Grosse SD, Krueger KV, Pike J. Estimated annual and lifetime labor productivity in the United States, 2016: Implications for economic evaluations. J Med Econ. Jun 2019;22(6):501-508. doi:10.1080/13696998.2018.1542520
43. US Bureau of Labor Statistics. Average hourly and weekly earnings of all employees on private nonfarm payrolls by industry sector, seasonally adjusted. Accessed 05/01/21, 2023. https://www.bls.gov/news.release/empsit.t19.htm
44. U.S. Catalog of age-and medical condition—specific healthcare costs Accessed April, 2025. https://uwchoice.shinyapps.io/futuremedicalcosts/
45. Prosser LA, Payne K, Rusinak D, Shi P, Uyeki TM, Messonnier ML. Valuing health across the lifespan: health state preferences for seasonal influenza illnesses in patients of different ages. Value in Health. 2011;14(1):135-43. doi:10.1016/j.jval.2010.10.026
46. Morrow AJ, Sykes R, McIntosh A, et al. A multisystem, cardio-renal investigation of post-COVID-19 illness. Nature Medicine. 2022/06/01 2022;28(6):1303-1313. doi:10.1038/s41591-022-01837-9
47. Cox IA, Arriagada NB, Graaff Bd, et al. Health-related quality of life of patients with idiopathic pulmonary fibrosis: a systematic review and meta-analysis. European Respiratory Review. 2020;29(158):200154. doi:10.1183/16000617.0154-2020
48. Cadham C, Rose A, Mercon K, Wittenberg E, Prosser L. Framing effects for response choices in a time trade off survey. presented at: Annual meeting of the Society for Medical Decision Making; October 2024 Boston, MA.
49. Arias E. United States life tables, 2017. Natl Vital Stat Rep. Jun 2019;68(7):1-66.
50. Janssen MF, Szende A, Cabases J, Ramos-Goñi JM, Vilagut G, König HH. Population norms for the EQ-5D-3L: A cross-country analysis of population surveys for 20 countries. Eur J Health Econ. Mar 2019;20(2):205-216. doi:10.1007/s10198-018-0955-5

© 2025 Prosser LA et al. *JAMA Network Open*.
