# Supplementary material for: Cost-Effectiveness of 2023-2024 COVID-19 Vaccination in US Adults
Source: JAMA Netw Open. 2025 Aug 7;8(8):e2523688. doi: 10.1001/jamanetworkopen.2025.23688 (PMC12332627; doi:10.1001/jamanetworkopen.2025.23688)
Supplement: Supplement 2. — eTable 1. Alternate Seasonality-Adjusted Vaccine Inputs by Disease Outcome, Age Group, and Vaccine Dose eTable 2. Disaggregated Disease Outcomes and Outcomes Averted for 1-Dose and 2-Dose Strategies, Per 100 000, Phase 2 Estimates eTable 3. Scenario Analyses, ICER ($/QALY Gained) eTable 4. Cost Per Cases Averted (Societal Perspective) and Number Needed to Vaccinate for 1-Dose and 2-Dose Strategies by Age Group and Type of Disease Outcome eFigure 1. Model Schematic eFigure 2. Derivation of Seasonality-Adjusted Vaccine Impact eFigure 3. Scenario Analysis: Vaccine Dose Cost eReferences. [file jamanetwopen-e2523688-s002.pdf]

Supplemental Online Content

Prosser LA, Wallace M, Rose AM, et al. Cost-effectiveness of 2023-2024 COVID-19 vaccination in US adults. *JAMA Netw Open*. 2025;8(8):e2523688. doi:10.1001/jamanetworkopen.2025.23688

eTable 1. Alternate Seasonality-Adjusted Vaccine Inputs by Disease Outcome, Age Group, and Vaccine Dose

eTable 2. Disaggregated Disease Outcomes and Outcomes Averted for 1-Dose and 2-Dose Strategies, Per 100 000, Phase 2 Estimates

eTable 3. Scenario Analyses, ICER ($/QALY Gained)

eTable 4. Cost Per Cases Averted (Societal Perspective) and Number Needed to Vaccinate for 1-Dose and 2-Dose Strategies by Age Group and Type of Disease Outcome

eFigure 1. Model Schematic

eFigure 2. Derivation of Seasonality-Adjusted Vaccine Impact

eFigure 3. Scenario Analysis: Vaccine Dose Cost

eReferences

This supplemental material has been provided by the authors to give readers additional information about their work.

© 2025 Prosser LA et al. *JAMA Network Open*.

Supplemental Materials, Phase 2

eTable 1. Alternate Seasonality-Adjusted Vaccine Impact Inputs by Disease Outcome, Age Group, and Vaccine Dose

Age	1-dose			2-dose			Source
	Base	Low	High	Base	Low	High	
**Alternate season #1: winter peak**							
Seasonality-adjusted vaccine impact, symptomatic illness and hospitalization							
18-49 y	0.306	0.104	0.442	0.437	0.176	0.511	[1-3], assumption[a]
50-64 y	0.323	0.113	0.451	0.442	0.181	0.515	
≥65 y	0.334	0.121	0.460	0.443	0.181	0.516	
Seasonality-adjusted vaccine impact, critical illness (hospitalization with ICU stay and death)							
18-49 y	0.411	0.212	0.657	0.538	0.375	0.660	[1,2], assumption[a]
50-64 y	0.426	0.229	0.657	0.542	0.380	0.662	
≥65 y	0.432	0.242	0.657	0.540	0.379	0.660	
**Alternate season #2: spring peak**							
Seasonality adjusted vaccine impact, symptomatic illness and hospitalization							
18-49 y	0.276	0.083	0.411	0.449	0.203	0.522	[1-3], assumption[a]
50-64 y	0.268	0.073	0.403	0.443	0.196	0.517	
≥65 y	0.269	0.070	0.401	0.439	0.194	0.514	
Seasonality adjusted vaccine impact, critical illness (hospitalization with ICU stay and death)							
18-49 y	0.441	0.195	0.674	0.574	0.416	0.690	[1,2], assumption[a]
50-64 y	0.440	0.188	0.672	0.569	0.409	0.686	
≥65 y	0.444	0.190	0.670	0.571	0.410	0.688	

ICU = Intensive care unit

[a] Assumed linear waning from the VE observed at 180 days until the end of the year.

© 2025 Prosser LA et al. *JAMA Network Open*.

eTable 2. Disaggregated Disease Outcomes and Outcomes Averted for 1-Dose and 2-Dose Strategies, Per 100 000, Phase 2 Estimates*

Age	Strategy	Disease Outcomes					Outcomes Averted				
		Cases	Hosp.	ICU	Adverse events	Deaths	Cases	Hosp.	ICU	Adverse events	Deaths
18-49 y	No 2023-2024 COVID-19 vaccination	31,450	44	5.4	NA	1.1	NA	NA	NA	NA	NA
	Vaccination, 1 dose (2023-2024 COVID-19 vaccine)	20,128	28	2.9	10,602	0.6	11,322	16	2.5	(10,602)	0.5
	Vaccination, 2 doses (2023-2024 COVID-19 vaccine)	17,895	25	2.5	21,203	0.5	2,233	3	0.5	(10,602)	0.1
50-64 y	No 2023-2024 COVID-19 vaccination	28,410	155	31.0	NA	7.6	NA	NA	NA	NA	NA
	Vaccination, 1 dose (2023-2024 COVID-19 vaccine)	18,268	100	16.8	10,600	4.3	10,142	55	14.2	(10,600)	3.3
	Vaccination, 2 doses (2023-2024 COVID-19 vaccine)	16,023	87	14.0	21,201	3.6	2,244	12	2.9	(10,600)	0.7
≥65 y	No 2023-2024 COVID-19 vaccination	33,390	790	113.8	NA	59.5	NA	NA	NA	NA	NA
	Vaccination, 1 dose (2023-2024 COVID-19 vaccine)	21,804	516	62.5	13,700	35.1	11,586	274	51.3	(13,700)	24.4
	Vaccination, 2 doses (2023-2024 COVID-19 vaccine)	18,899	447	51.3	27,401	29.5	2,905	69	11.1	(13,700)	5.6

*Cases include all cases of COVID-19 illness: non-medically attended illnesses, medically-attended illnesses, non-hospitalized (outpatient and emergency department), hospitalizations, and deaths.

© 2025 Prosser LA et al. *JAMA Network Open*.

eTable 3. Scenario Analyses, ICER ($/QALY Gained)

	Strategy	
	1-dose	2-dose
**Base case**		
18-49 y	$163,255	$1,317,714
50-64 y	$80,427	$777,612
≥65 y	$11,936	$255,122
**Alternate seasonality scenario: winter peak**		
18-49 y	$201,455	$607,744
50-64 y	$96,028	$483,429
≥65 y	$14,788	$198,802
**Alternate seasonality scenario: spring peak**		
18-49 y	$224,209	$439,252
50-64 y	$117,080	$315,145
≥65 y	$25,126	$115,650
**Seasonality-adjusted vaccine impact scenarios**		
**Scenario 1: Vaccine impact set to lower bounds**		
18-49 y	$505,148	$3,377,607
50-64 y	$278,403	$1,322,035
≥65 y	$94,739	$370,897
**Scenario 2: Vaccine impact set to upper bounds**		
18-49 y	$108,377	$6,594,605
50-64 y	$36,230	$4,519,196
≥65 y	CS	$1,280,380
**Scenario 3: Vaccine impact against hospitalization applied to all endpoints**		
18-49 y	$168,932	$1,342,797
50-64 y	$93,020	$822,061
≥65 y	$17,410	$269,781
**Scenario 4: Vaccine impact against critical illness applied to all endpoints**		
18-49 y	$119,338	$1,069,740
50-64 y	$54,576	$672,042
≥65 y	CS	$229,468
**Probability of symptomatic illness, non-hospitalized**		
**0.1**		
18-49 y	$504,919	$6,904,206
50-64 y	$175,920	$1,616,648
≥65 y	$27,384	$365,420
**0.2**		
18-49 y	$264,579	$2,337,532

© 2025 Prosser LA et al. *JAMA Network Open*.

50-64 y	$111,693	$1,024,872
≥65 y	$19,725	$309,485
**0.3**		
18-49 y	$172,139	$1,395,606
50-64 y	$75,820	$743,109
≥65 y	$13,698	$267,206
**0.4**		
18-49 y	$123,199	$988,908
50-64 y	$52,920	$578,315
≥65 y	$8,830	$234,125
**0.5**		
18-49 y	$92,903	$762,176
50-64 y	$37,034	$470,173
≥65 y	$4,817	$207,536
**Probability of hospitalization**		
**¼ base case**		
18-49 y	$194,430	$1,501,728
50-64 y	$164,173	$1,207,262
≥65 y	$93,904	$624,028
**½ base case**		
18-49 y	$183,347	$1,435,441
50-64 y	$129,533	$1,024,868
≥65 y	$52,541	$433,533
**2x base case**		
18-49 y	$129,752	$1,128,142
50-64 y	$23,416	$506,001
≥65 y	CS	$120,341
**3x base case**		
18-49 y	$102,935	$982,164
50-64 y	CS	$359,910
≥65 y	CS	$64,599
**4x base case**		
18-49 y	$80,985	$866,292
50-64 y	CS	$268,659
≥65 y	CS	$34,133
**Probability of critical illness**		
**2x base case**		
18-49 y	$140,285	$1,174,541
50-64 y	$39,801	$555,268
≥65 y	CS	$167,809

© 2025 Prosser LA et al. *JAMA Network Open*.

**3x base case**		
18-49 y	$121,002	$1,057,636
50-64 y	$14,969	$423,809
≥65 y	CS	$120,583
**4x base case**		
18-49 y	$104,587	$960,376
50-64 y	CS	$336,967
≥65 y	CS	$90,993
**Probability of hospitalization, critical care, and death**		
**All low**		
18-49 y	$198,847	$1,532,966
50-64 y	$173,150	$1,266,927
≥65 y	$94,352	$641,049
**All high**		
18-49 y	$42,634	$626,452
50-64 y	CS	$266,650
≥65 y	CS	$59,219
**Probability of hospitalization**		
**All low**		
18-49 y	$219,309	$1,721,802
50-64 y	$193,040	$1,431,978
≥65 y	$110,879	$752,024
**All high**		
18-49 y	$63,501	$749,804
50-64 y	CS	$326,446
≥65 y	CS	$73,208
**Vaccine setting**		
**100% pharmacy**		
18-49 y	$153,774	$1,256,462
50-64 y	$73,296	$739,111
≥65 y	$8,746	$240,613
**100% physician office**		
18-49 y	$191,677	$1,501,322
50-64 y	$101,802	$893,021
≥65 y	$21,497	$298,615
**100% mass vaccination**		
18-49 y	$151,556	$1,242,135
50-64 y	$71,628	$730,106
≥65 y	$8,000	$237,220
**5- or 7-month interval between doses**		

© 2025 Prosser LA et al. *JAMA Network Open*.

18-49 y	$163,255	$1,444,227
50-64 y	$80,427	$888,173
≥65 y	$11,936	$278,909
**Vaccination related costs**		
**All low**		
18-49 y	$22,318	$407,248
50-64 y	CS	$198,125
≥65 y	CS	$50,768
**All high**		
18-49 y	$285,142	$2,105,115
50-64 y	$172,094	$1,272,543
≥65 y	$53,251	$443,059
**Vaccine dose cost**		
**$20**		
18-49 y	$43,744	$545,664
50-64 y	CS	$292,330
≥65 y	CS	$72,240
**$30**		
18-49 y	$55,696	$622,869
50-64 y	CS	$340,858
≥65 y	CS	$90,529
**$40**		
18-49 y	$67,647	$700,074
50-64 y	$8,523	$389,386
≥65 y	CS	$108,817
**$50**		
18-49 y	$79,598	$777,279
50-64 y	$17,511	$437,914
≥65 y	CS	$127,105
**$60**		
18-49 y	$91,549	$854,484
50-64 y	$26,499	$486,442
≥65 y	CS	$145,393
**$70**		
18-49 y	$103,500	$931,689
50-64 y	$35,487	$534,971
≥65 y	CS	$163,681
**$80**		
18-49 y	$115,451	$1,008,894
50-64 y	$44,475	$583,499

© 2025 Prosser LA et al. *JAMA Network Open*.

≥65 y	CS	$181,970
**$90**		
18-49 y	$127,402	$1,086,099
50-64 y	$53,463	$632,027
≥65 y	CS	$200,258
**$100**		
18-49 y	$139,353	$1,163,304
50-64 y	$62,451	$680,555
≥65 y	$3,895	$218,546
**$110**		
18-49 y	$151,304	$1,240,509
50-64 y	$71,439	$729,083
≥65 y	$7,915	$236,834
**$120-base case**		
18-49 y	$163,255	$1,317,714
50-64 y	$80,427	$777,612
≥65 y	$11,936	$255,122

ICER = Incremental cost-effectiveness ratio; QALY = quality-adjusted life year; critical illness = includes hospitalizations with intensive care unit stays and/or ventilator use, or death; CS = cost savings

© 2025 Prosser LA et al. *JAMA Network Open*.

eTable 4. Cost Per Cases Averted (Societal Perspective) and Number Needed to Vaccinate for 1-Dose and 2-Dose Strategies by Age Group and Type of Disease Outcome

Age group	Strategy	$/disease outcome averted			Number needed to vaccinate to avert 1:		
		Case	Hosp	Death	Case	Hosp	Death
18-49 y	Vaccination, 1 dose (2023-2024 COVID-19 vaccine)	$1,207	$856,552	$28,187,357	9	6,270	206,345
	Vaccination, 2 doses (2023-2024 COVID-19 vaccine)	$7,644	$5,426,426	$190,514,465	45	31,793	1,116,226
50-64 y	Vaccination, 1 dose (2023-2024 COVID-19 vaccine)	$882	$161,710	$2,685,832	10	1,807	30,015
	Vaccination, 2 doses (2023-2024 COVID-19 vaccine)	$7,140	$1,308,609	$23,542,054	45	8,167	146,918
≥65 y	Vaccination, 1 dose (2023-2024 COVID-19 vaccine)	$256	$10,830	$121,913	9	365	4,107
	Vaccination, 2 doses (2023-2024 COVID-19 vaccine)	$4,802	$202,970	$2,506,208	34	1,455	17,966

© 2025 Prosser LA et al. *JAMA Network Open*.

eFigure 1. Model Schematic

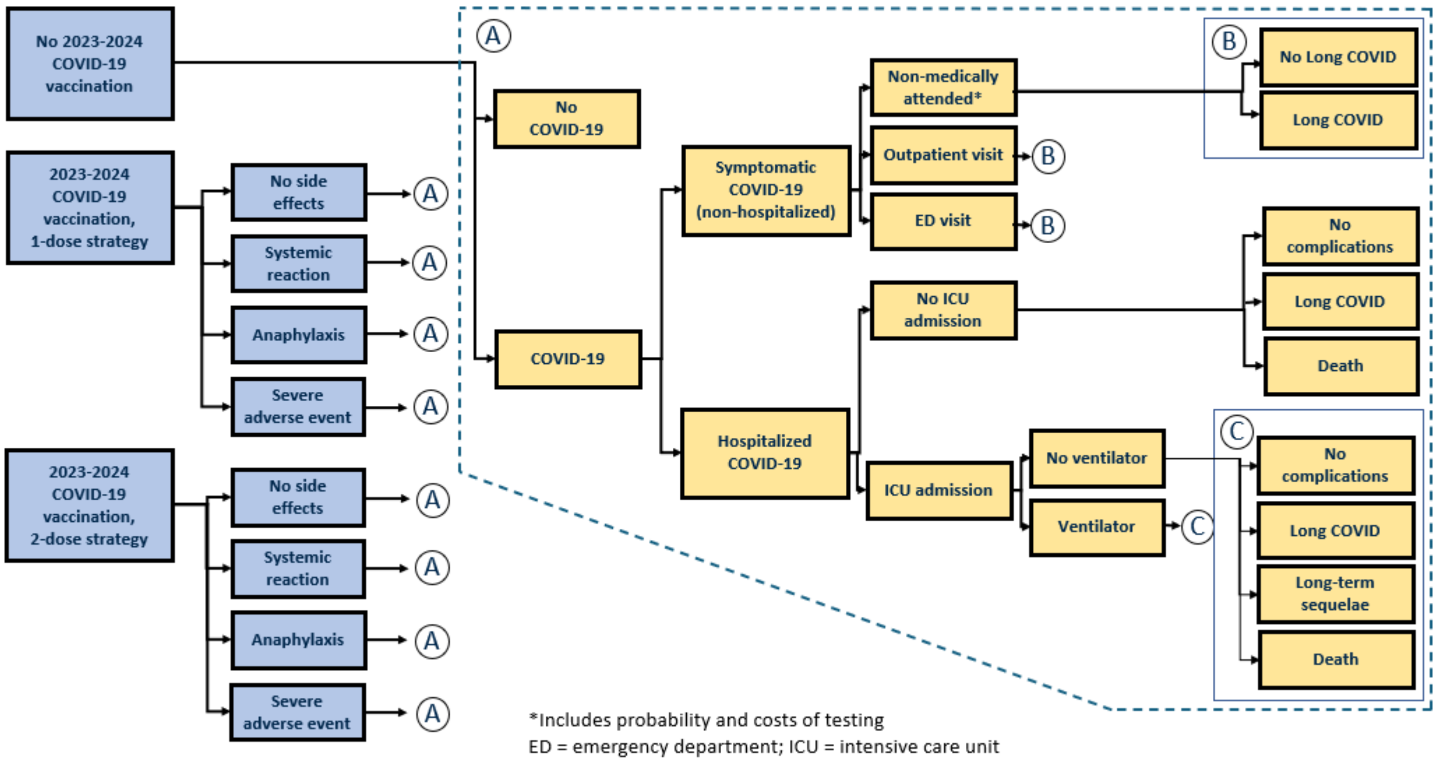

*Includes probability and costs of testing
ED = emergency department; ICU = intensive care unit

© 2025 Prosser LA et al. *JAMA Network Open*.

eFigure 2. Derivation of Seasonality-Adjusted Vaccine Impact

Data for vaccine effectiveness (VE) of the COVID-19 bivalent booster vaccine was sourced from two networks: VISION Vaccine Effectiveness (VISION) and Investigating Respiratory Viruses in the Acutely Ill (IVY). [2,3] Using data from both the VISION and IVY studies, we calculated area under the curve to estimate VE for a 2023-2024 mRNA vaccine dose over a 12-month period. Using data available for 180 days following vaccination, 3 scenarios were used to extrapolate VE to the one year period: an optimistic scenario which assumed the VE observed at 180 days remained the same for the duration of the year, a conservative scenario in which VE waned immediately to zero at 181 days and for the remainder of the year, and a midpoint scenario which assumed linear waning from the VE observed at 180 days until the end of the year. The minimum of the conservative estimates and maximum of the optimistic estimates were selected for the lower and upper bounds for VE. Data were unavailable to inform VE against symptomatic illness.

Seasonality-adjusted vaccine impact for two alternative COVID-19 seasons were also considered one with a higher peak in the winter and a second alternate with a peak in the spring. (Table 1)
A second dose of the 2023-2024 COVID-19 vaccine was assumed to be given at 6 months after the first dose with vaccine effectiveness assumed to follow initial vaccine effectiveness observed in the VISION and IVY studies and follow the same waning pattern. [2,3] We calculated area under the curve to estimate seasonality-adjusted vaccine impact over a 12-month period for a 2-dose 2023-2024 mRNA vaccine series.

a. Vaccine effectiveness

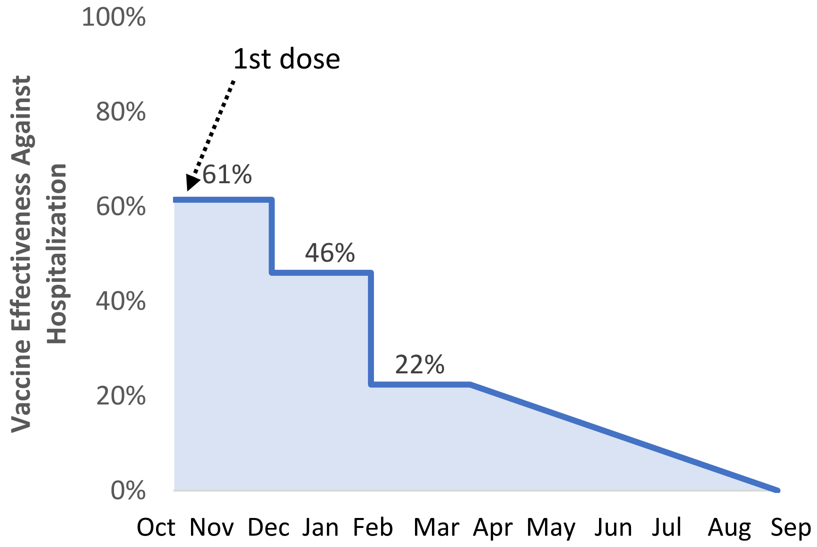

© 2025 Prosser LA et al. *JAMA Network Open*.

b. Seasonality-adjusted vaccine impact, 1-dose strategy

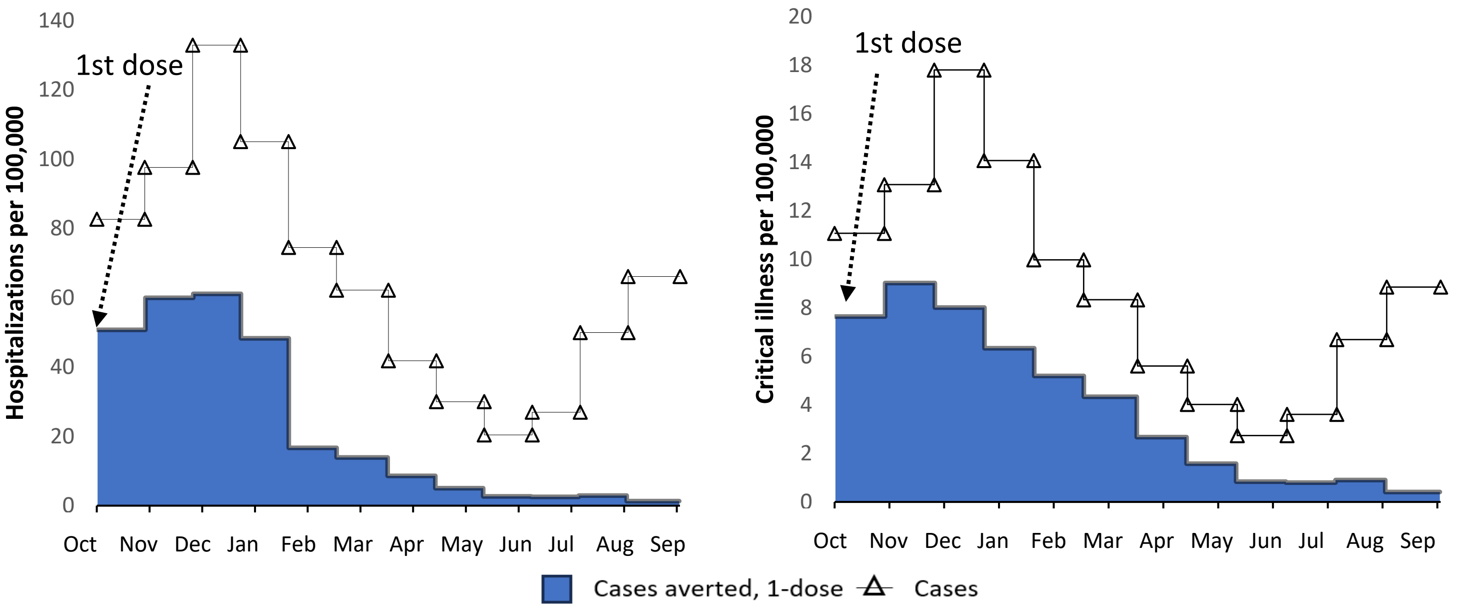

c. Seasonality-adjusted vaccine impact, 2-dose strategy

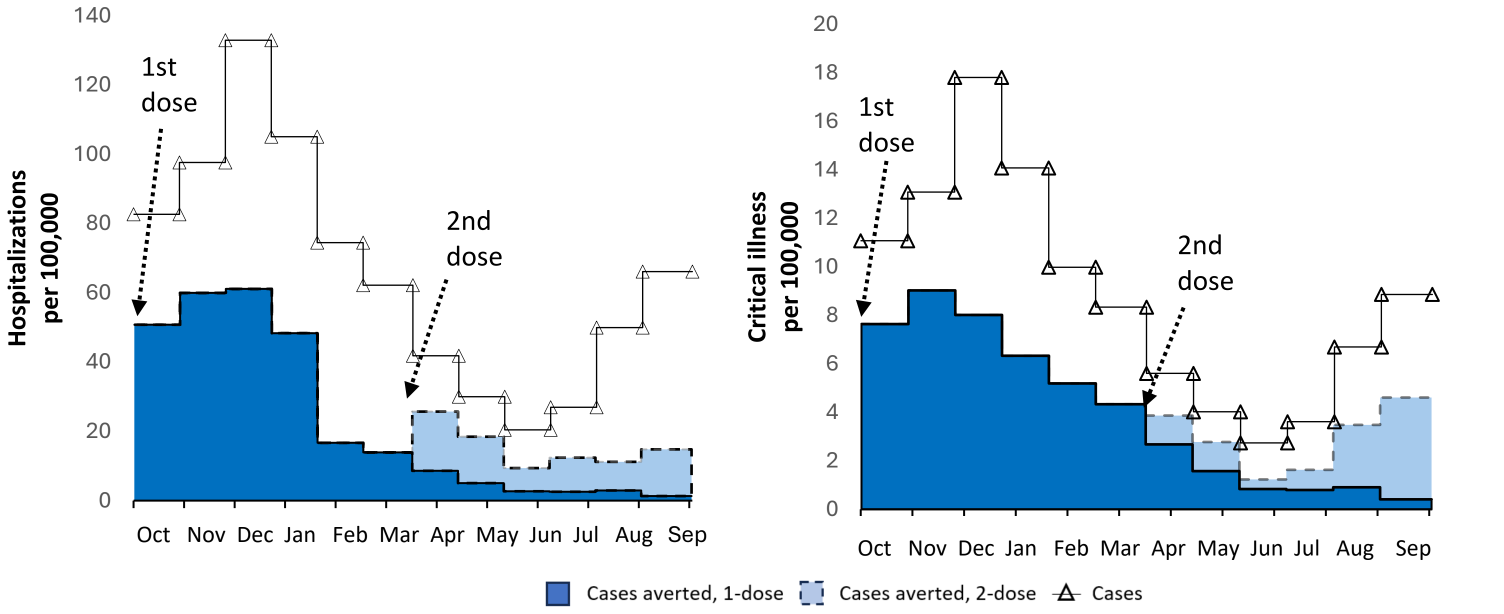

© 2025 Prosser LA et al. *JAMA Network Open*.

eFigure 3. Scenario Analysis: Vaccine Dose Cost

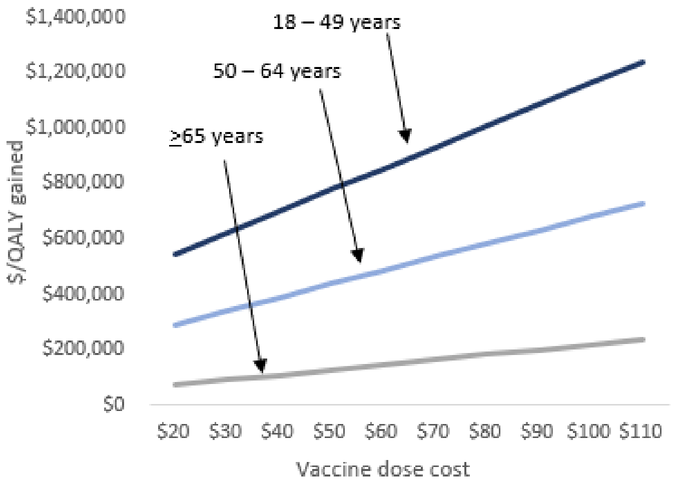

© 2025 Prosser LA et al. *JAMA Network Open*.

eReferences

1. COVID-NET, Oct 2022-Sept 2023. https://www.cdc.gov/covid/php/covid-net/index.html
2. Link-Gelles R. Monovalent and bivalent VE, against hospitalization and critical illness by Omicron subvariant in adults ≥18 years, VISION Network. U.S. Centers for Disease Control and Prevention. https://www.cdc.gov/acip/downloads/slides-2023-06-21-23/02-COVID-Havers-Galang-Link-Gelles-508.pdf
3. Link-Gelles R. Monovalent and bivalent VE against hospitalization among adults aged ≥18 years, IVY Network. U.S. Centers for Disease Control and Prevention. https://www.cdc.gov/acip/downloads/slides-2023-06-21-23/02-COVID-Havers-Galang-Link-Gelles-508.pdf

© 2025 Prosser LA et al. *JAMA Network Open*.
