# Supplementary material for: Cost-Effectiveness of 2023-2024 COVID-19 Vaccination in US Adults
Source: JAMA Netw Open. 2025 Aug 7;8(8):e2523688. doi: 10.1001/jamanetworkopen.2025.23688 (PMC12332627; doi:10.1001/jamanetworkopen.2025.23688)
Supplement: Supplement 3. — Data Sharing Statement [file jamanetwopen-e2523688-s003.pdf]

# Data Sharing Statement

Prosser. Cost-Effectiveness of COVID-19 Vaccination in 2023 to 2024 in US Adults. *JAMA Netw Open*. Published August 07, 2025. doi:10.1001/jamanetworkopen.2025.23688

**Data**
**Data available:** Yes
**Data types:** Data (not involving human participants)
**How to access data:** All inputs for the model are available in the supplement
**When available:** With publication

**Supporting Documents**
**Document types:** Other (please specify)
**Additional Information:** All inputs for the model are available in the supplement
**How to access documents:** All inputs for the model are available in the supplement
**When available:** With publication

**Additional Information**
**Who can access the data:** All inputs for the model are available in the supplement
**Types of analyses:** All inputs for the model are available in the supplement
**Mechanisms of data availability:** All inputs for the model are available in the supplement
